# A Novel Approach to Selectively Target Neuronal Subpopulations Reveals Genetic Pathways That Regulate Tangential Migration in the Vertebrate Hindbrain

**DOI:** 10.1371/journal.pgen.1002099

**Published:** 2011-06-16

**Authors:** Karsten Benzing, Stefanie Flunkert, Andreas Schedl, Dieter Engelkamp

**Affiliations:** 1 Max Planck Institute for Brain Research, Department of Neuroanatomy, Frankfurt, Germany; 2 INSERM UMR636, Centre de Biochimie, Nice, France; 3 University of Nice Sophia Antipolis, Nice, France; 4 Department of Biology, Friedrich-Alexander-University of Erlangen-Nürnberg, Erlangen, Germany; University of Toronto and Samuel Lunenfeld Research Institute at Mount Sinai Hospital, Canada

## Abstract

Vertebrate genes often play functionally distinct roles in different subsets of cells; however, tools to study the cell-specific function of gene products are poorly developed. Therefore, we have established a novel mouse model that enables the visualization and manipulation of defined subpopulations of neurons. To demonstrate the power of our system, we dissected genetic cascades in which *Pax6* is central to control tangentially migrating neurons of the mouse brainstem. Several *Pax6* downstream genes were identified and their function was analyzed by over-expression and knock-down experiments. One of these, *Pou4f2*, induces a prolonged midline arrest of growth cones to influence the proportion of ipsilaterally versus contralaterally settling neurons. These results demonstrate that our approach serves as a versatile tool to study the function of genes involved in cell migration, axonal pathfinding, and patterning processes. Our model will also serve as a general tool to specifically over-express any gene in a defined subpopulation of neurons and should easily be adapted to a wide range of applications.

## Author Summary

In mammals, many genes execute a unique set of distinctive and common functions in different cell types. Strategies to address these individual roles often involve the generation of series of transgenic animals. Here, we present a novel approach that combines a single transgenic mouse line with tissue-specific transfection protocols and organotypic cultures to enable the quick analysis of numerous genes in a cell-specific manner. As a proof of principle, we analyzed the function of transcription factors in tangentially migrating neurons of the developing vertebrate hindbrain. We identified a temporary halt in migration as a novel mechanism for neurons to decide whether to cross or not cross the midline. Our model may serve as a general tool to quickly study axonal pathfinding, neuronal cell migration, or patterning processes in a well-defined population of neurons.

## Introduction

Understanding cell-specific regulatory mechanisms is a major challenge in the post-genome era. Particularly in mammals, the reiterated usage of the same transcription factor in distinct subsets of cells or during distinct developmental time points provides the basis to generate thousands of individual cell types with a relatively small number of genes. A single transcription factor may therefore elicit variable downstream effects depending on the context of its expression. Tissue-specific knockout strategies based e.g. on the Cre-lox-system, or promoter-driven transgenic models allow a cell-specific manipulation of genes. However, as these techniques rely on the generation of new transgenic animals for each gene-combination analyzed they are laborious and time-consuming. Here, we combined a transgenic model with tissue-specific transfection protocols and organotypic cultures to enable the quick analysis of numerous genes in a cell-specific manner. As a proof of principle we applied our system to decode molecular pathways initiated by the transcription factor *Pax6* which is involved in neuronal cell migration and axonal pathfinding processes.

*Pax6*, a homeodomain and paired domain containing transcription factor, is a major determinant of visual and olfactory sensory structures and is essential for a variety of patterning and pathfinding processes throughout the nervous system [1]–[3]. Depending on the context and area of expression *Pax6* initiates varying downstream effects. Homozygous *small eye* (*Pax6^Sey/Sey^*) mouse and rat embryos, which lack functional *Pax6*, do neither generate eye nor nasal structures and are deficient in ventral diencephalic structures [4]–[9]. In the ventral hindbrain and spinal cord, *Pax6* controls the dorso-ventral patterning of motorneurons and of interneurons [5], [10]. In the cerebral cortex *Pax6* determines the neurogenic potential of radial glial cells [11], [12]. Throughout the developing nervous system, with the exception of the midbrain, *Pax6* is expressed in a ventral and a dorsal pool of progenitor cells. Although the dorsal *Pax6* expression domain has achieved much less attention than the ventral domain there is evidence that *Pax6* plays a pivotal role in the specification and migration of neurons derived from this domain [13]–[16].

**Figure 1:**
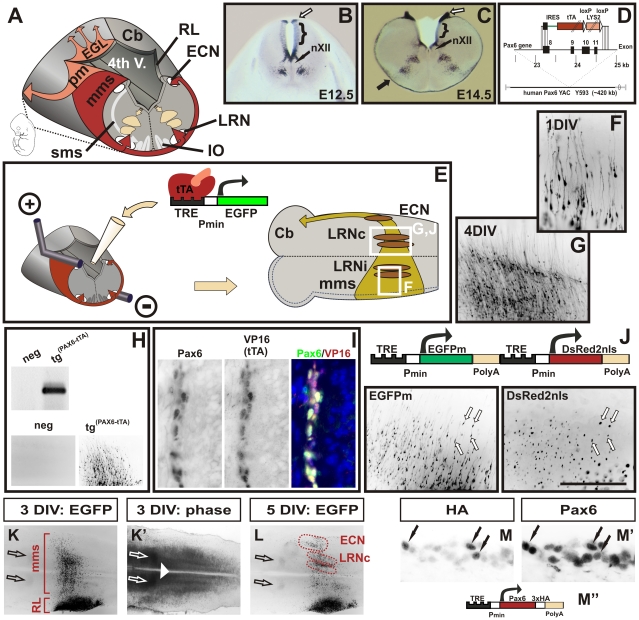
A novel mouse model to visualize and manipulate Pax6 positive neurons. (A) Schematic diagram of tangentially migrating neurons in the mouse brainstem. *Pax6* positive neurons of the marginal migration stream (*mms*) and of the pontine migration (*pm*) are derived from the rhombic lip (RL) and generate the lateral reticular (LRN), the external cuneate nuclei (ECN), the pontine nuclei and the pontine reticulotegmental nuclei (not shown), respectively. *Pax6* positive external granule cells (EGL) initially migrate rostrally on the surface of the cerebellar primordium (Cb). *Pax6* negative neurons of the submarginal migration stream (*sms*) generate the inferior olive (IO). Distribution of *Pax6* positive cells in the wt hindbrain at E12.5 (B) and at E14.5 (C) as shown by in situ hybridization on coronal vibratome sections. *Pax6* is expressed in precursor cells at the caudal RL (open arrows in B,C), in ventral precursor cells at the basal plate, in groups of ventrally migrating neurons and in the *mms* (filled arrow in C). The dorsal alar plate (brackets in B,C), the hypoglossal nuclei (nXII), and the floor plate are negative for *Pax6*. (D) Diagram of the targeting construct to replace the coding sequence of human PAX6 in YAC Y593 with an IRES-tau-tTA-LYS2 cassette. For a more detailed description of the construct see the Materials and Methods section. (E) Schematic drawing of the electroporation and brainstem culture procedures. Responder constructs consisting of tetracycline responsive elements (TRE), a minimal promoter (p_min_), and a reporter gene (e.g. EGFP) are injected into the fourth ventricle of E12.5 *Tg^(PAX6-tTA)^* embryos and then transfected into one RL by electroporation. Subsequently, the hindbrain is dissected and cultured with the ventricular face onto MilliporeCM filters. After prolonged cultures *mms* neurons settle either in the ipsi- or the contralateral LRN (LRNi, LRNc), or in the ECN. All neurons project to the contralateral cerebellar hemisphere (Cb). (F and G) Migrating *mms* neurons visualized by EGFP fluorescence in transfected brainstems. The enlarged areas shown are indicated by white boxes in (E). After 1 day in vitro culture (1 DIV) migrating neurons with long leading and short trailing processes emerge from the transfected RL (F). After 4DIV the majority of *mms* neurons have settled in contralateral LRN (LRNc) (G). Genotyping of transfected cultures confirms that responder constructs are exclusively activated in *Tg^(PAX6-tTA)^* and not in non-transgenic embryos (H). Double-immunolabeling of *mms* neurons with *Pax6* and VP16 (to recognize tTA) antibodies confirm a complete overlap of *Pax6* and tTA expression (I). A responder vector containing two TRE elements drives expression of two genes simultaneously: a cytoplasmic green fluorescent protein (EGFPm) and a nuclear red fluorescent protein (DsRed2nls) (J). The left and right images are green and red fluorescent images, respectively, of migrating *mms* neurons after 1.5DIV. K, K′, and L are low magnification fluorescence (K, L) and phase contrast (K′) images of cultures transfected with an EGFP construct as indicated in E. After 3DIV several hundred neurons have left the transfected rhombic lip (RL) to migrate along the *mms*. At 5DIV all neurons have reached their target positions. 25 similar cultures were used to outline the territories of the LRNc and ECN in respect to surrounding “landmarks”: the superior olivary nuclei (open arrows), the inferior olivary nuclei, the midline (white arrowhead), and the rhombic lip (RL). Double-immunolabeling of cultures transfected with a hemagglutinin (HA)-tagged *Pax6* construct (M, M′, M'') confirm that over-expression results in *Pax6* levels comparable to endogenous *Pax6* levels. Immunolabeling with HA-tag antibodies (M) mark transfected cells (black arrows) which express endogenous and exogenous *Pax6*. Note, that *Pax6* levels in some untransfected cells (only endogenous *Pax6*) are as high as *Pax6* levels in transfected cells (endogenous plus exogenous *Pax6*). (The scale bar represents 0.8 mm in [B]; 1 mm in [C]; 150 µm in [F]; 0.44mm in [G, J]; 0.9 mm in [H]; 70 µm in [I]; 1.8 mm in [K, K′, L]; 50 µm in [M, M′]).

The dorsal domain of *Pax6* positive neuronal precursors of the hindbrain includes the rhombic lip (RL) [14], [16] which comprises the interface between the dorsal neuroepithelium and the roof plate. The RL is the source of several tangentially migrating neurons (see also Figure 1A) [14], [17]–[24]. The most notable are the neurons of the marginal migratory stream (*mms*; also *pes*) which migrate from the rhombic lip circumferentially around the medulla towards their contralateral destinations to settle in the ECN (external cuneate nuclei) and the LRN (lateral reticular nuclei) [17], [25]. Owing to the superficial nature of the *mms* migration these neurons serve as paradigm to study neuronal migration and axonal pathfinding processes.

The highly complex neuronal circuits of the vertebrate nervous system are established during development when growing axons travel considerable distances towards their targets to generate the appropriate connections. This wiring process depends on attractive and repulsive factors which emanate from final or intermediate cellular targets and which are interpreted by cell surface receptors located on axonal growth cones [26], [27]. Although the general principles were uncovered during the past years our understanding of axonal pathfinding processes is far from being complete. Current methods to analyze candidates regulating neuronal migration and axonal navigation processes are laborious and often involve the generation of transgenic animals for each gene analyzed. Non-transgenic methods, as DiI labeling of neurons or vector-driven mis-expression of gene constructs, are suitable for use with certain applications, however, they are neither cell specific nor can they be targeted to distinct neuronal subpopulations.

Here we describe a novel transgenic mouse model, which allows the specific and exclusive visualization and manipulation of subsets of neurons in the developing brain. To demonstrate the power of this system we have analyzed the role of *Pax6* in migrating neurons of the brainstem. In *Pax6* mutant mice migration of these neurons is distorted and some neurons differentiate at ectopic positions. Using transplantation, knock-down and over-expression experiments we show that distinct migratory features are controlled by discrete sets of *Pax6* downstream genes. These results demonstrate the potential of our transgenic mouse model as a tool to study the role of *Pax6* in individual neurons. Moreover, our system should be widely applicable to study virtually any gene that acts during cell determination, axonal pathfinding and/ or cell migration processes.

## Results

### A novel mouse model to visualize and manipulate subsets of neurons

The functional analysis of genes in restricted tissues often involves the generation of inducible knockout mice or mice over expressing transgenic constructs. To simplify this time-consuming process we developed an in vitro model that enables the visualization and manipulation of defined populations of neurons. To label neurons in a largely unlabelled background we searched for genes that were expressed in only a subset of neuronal precursors and in migrating neurons. *Pax6* meets these criteria ideally. *Pax6* is expressed in several groups of tangentially migrating neurons and their precursors as well as in a small population of radially migrating neurons and their precursors (Figure 1A–1C) [5], [10], [12], [14], [16], [28].

We adopted the Tet binary system [29] and generated YAC (yeast artificial chromosome) transgenic mice which expressed the tetracycline dependent transactivator (tTA) in all *Pax6* positive cells. A 420 kb YAC spanning the human PAX6 locus (Y593) [30] was modified such that the PAX6 coding region was replaced with a cassette containing an IRES (internal ribosomal entry site) and the tTA (Figure 1D). Previously, we and others had shown that the unmodified YAC Y593 contains all elements driving full functional PAX6 expression [30]–[32] and, in agreement with this, *Tg^(PAX6-tTA)^* mice showed a wide overlap of tTA and endogenous murine *Pax6* expression (Figure S1). *Tg^(PAX6-tTA)^* mice were entirely normal and control experiments insured that neuronal patterning and migration was unaltered.

tTA is a transcriptional activator that at moderate levels of expression is completely inert in vertebrates, yet, enables the activation of artificial constructs containing a tTA-DNA-binding element (TRE  =  tetracyline responsive element). To examine whether our transgenic model specifically allows the labeling of only *Pax6* positive cells we introduced by electroporation reporter constructs driving the green fluorescent protein into transgenic embryos. In all instances only *Pax6* positive cells, e.g. retinal precursor cells, cortical precursors, or cerebellar granule cells, expressed the reporter genes (Figure S1). Non-transgenic embryos or *Pax6* negative tissues did not induce reporter gene expression (Figure 1H, Figure S1). Together these results demonstrate that *Tg^(PAX6-tTA)^* mice enable the targeting of reporter gene constructs specifically to *Pax6* positive cells and tissues during development.

As *Tg^(PAX6-tTA)^* mice allow any gene to be targeted to *Pax6* expressing cells, they are of potential value to study neuronal migration and axonal pathfinding processes and for the analysis of *Pax6* downstream effects. As a proof of principle, we chose to focus on the marginal migration stream (*mms*). Like other tangentially migrating neurons, *mms* neurons use the same or similar navigational cues as do growing axons, and migration of *mms* neurons is severely disturbed in *Pax6* mutant *Pax6^Sey/Sey^* mice (see below). Neurons of the *mms* are generated at the rhombic lip and migrate circumferentially around the embryonic brainstem to generate the contralateral lateral reticular (LRN) and the external cuneate (ECN) nuclei (Figure 1A) [16], [21]–[23], [25]. Migration starts at E13.0 and is completed by E16.5. *Pax6* is expressed in precursors at the rhombic lip, in all migrating neurons of the *mms* and during the initial period of settling in the target nuclei (Figure 1B, 1C and data not shown). Antibody staining and in situ hybridization (not shown) of *Tg^(PAX6-tTA)^* mice confirmed a complete overlap of *Pax6* and tTA expression in these neurons (Figure 1I).

To visualize migrating *mms* neurons in *Tg^(PAX6-tTA)^* mice, reporter constructs were introduced into neuronal precursor cells in the left rhombic lip by electroporation at E12.5 before migration had begun (Figure 1E). Whole brainstems including the cerebellar primordium were then sustained in organotypic filter cultures for up to 14 days as an open book preparation which allowed the observation of migrating neurons with a fluorescence microscope from above (Figure 1F, 1G). Our approach to use a binary system ensured that only *Pax6* positive neurons containing tTA and a TRE reporter construct expressed the desired reporter genes. This procedure resulted in the specific labeling of *mms* neurons originating only from one rhombic lip. *Pax6* positive neurons originating from the opposite rhombic lip remained unlabelled as were *Pax6* negative (and therefore tTA negative) neurons originating from regions close to the rhombic lip. Unlabelled neurons included neurons of the submarginal migration stream (*sms*) which generate the inferior olive (IO) thus demonstrating the specificity of our model. To allow the simultaneous visualization and manipulation of neurons we designed reporter constructs containing two TRE elements (Figure 1J). Control constructs co-expressed a cytoplasmic green fluorescent protein (EGFPm) and a nuclear red fluorescent protein (DsRed2nls) in 99% (±1; n = 10) of labeled neurons demonstrating that our reporter constructs enable the co-expression of two genes in the same neurons (Figure 1J). To enable statistical analysis of the cultures, the territories of the LRN and the ECN were delineated using visible landmarks (Figure 1K, 1K′, 1L; see also the Materials and Methods section). Immunolabeling of cultures expressing a HA-tagged Pax6 construct demonstrate that over-expression of TRE constructs in *Tg^(PAX6-tTA)^* transgenic cultures result in moderate levels of protein expression that are in the range of physiological Pax6 concentrations (Figure 1M, 1M′, 1M'').

### *Pax6* plays multiple roles in patterning and guiding migrating ECN and LRN neurons

**Figure 2:**
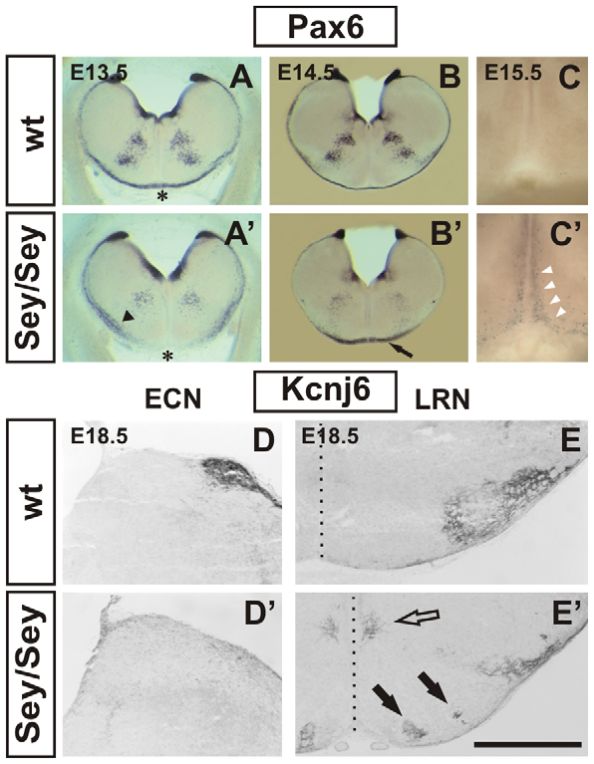
Migration defects of *Pax6^Sey/Sey^* pre-cerebellar neurons: histology. Coronal vibratome *Pax6* in situ hybridizations of wt (A - C) and mutant (A′–C′) embryos. The *Pax6^Sey/Sey^* mutation is a result of single base pair substitution in the *Pax6* gene that leads to a shortened non-functional protein product [4]. *Pax6* transcript levels, however, are largely unaffected in mutants therefore allowing the detection of *Pax6* expressing cells in *Pax6^Sey/Sey^* embryos. At E12.5 the *mms* has not yet formed (see Figure. 1B), but, at E13.5 first neurons of the *mms* have already crossed the midline (asterisk in A). In the mutant, migration of *mms* neurons is delayed, some neurons use a sub-marginal migration (arrowhead in A′), and by E13.5 neurons have not reached the midline (asterisk in A′). At E14.5 Pax6 positive neurons accumulate around the ventral midline (arrow in B′) and at E15.5 some neurons migrate dorsally along the ventral midline (white arrowheads in C′). In wt embryos all *mms* neurons migrate along the marginal migration route (A,B) and no *Pax6* positive cells are seen at the ventral midline (C). Immunohistochemical labeling with an α-Kcnj6 antibody on coronal E18.5 wt (D,E) and *Pax6^Sey/Sey^* (D′,E′) sections. The α-Kcnj6 antibody strongly labels the wt ECN (D) and the LRN (E). α-Kcnj6 is completely absent from the ECN territory in *Pax6^Sey/Sey^* embryos (D′) and mutant LRN neurons (E′) are scattered at the LRN territory, within the inferior olivary complex (black arrows in E′), and at both sides of the midline (open arrow in E′). The dotted line in E and E′ indicates the midline. (Scale bar is 1 mm in [A, A′]; 1.2 mm in [B, B′]; 380 µm in [C]; 0.55 mm in [D, D′, E, E′].)

*Pax6* mutant *Pax6^Sey/Sey^* mice display multiple neuronal patterning and migration defects. We therefore wished to determine whether *Pax6* also regulates the *mms*. At the anatomical level, several features of the *mms* are severely disturbed in *Pax6* mutant *Pax6^Sey/Sey^* embryos. Most noticeable, the initiation of migration and the midline crossing was delayed by 0.5 days (asterisks in Figure 2A, 2A′ and data not shown; see also Figure S2 and Figure 4A, 4A′ The expression patterns of *Pax2, Dcx, NK1R*, and *DopH* was unaltered indicating that there is no general developmental delay in the mutant brainstem (data not shown). In *Pax6^Sey/Sey^* embryos some migrating *mms* neurons used a sub-marginal instead of a marginal migration path (black arrowhead in Figure 2A′; see also Figure S2 and Figure 4A′) and at E14.5 a large number of mutant neurons accumulated around the midline suggesting a reduced pace in midline crossing (black arrow in Figure 2B, 2B′). Furthermore, a subset of *Pax6* positive neurons migrated along the midline into the parenchyma of the hindbrain (white arrowheads in Figure 2C, 2C′). We used a variety of markers, e.g. antibodies against the potassium channel *Kcnj6*, and DiI tracing of mossy fiber projections to discriminate between *mms* neurons (generating the ECN and LRN) and neurons of the *sms* (generating the IO). These experiments all indicated a complete loss of neurons in the ECN and a disorganized settling of neurons in the LRN (Figure 2D, 2D′, 2E, 2E′; Figure S2, and data not shown). Many *Kcnj6* positive neurons were even observed within the inferior olivary territory (black arrows in Figure 2E′) and dorsally to the IO at the midline (open arrow in Figure 2E′). In agreement with previous reports [16], we found a slight enlargement of the IO at E14.5 when we used the Ets transcription factor *Etv1* as a IO specific marker [33] (Figure S3 and data not shown). However, by labeling for the axon-guidance-molecule B (RgmB) no alterations in the general architecture of the IO were seen [34] (Figure S3). This is consistent with our observation that misguided *Pax6^Sey/Sey^* *mms* neurons make only a negligible contribution to the IO or settle in the periphery of the IO. In summary, these data demonstrate that migration of *Pax6^Sey/Sey^* *mms* neurons is severely disrupted. Mutant neurons of the *mms* are initially delayed. Later, a number of neurons use a sub-marginal migration path, migration is disturbed at the midline and several neurons migrate to ectopic positions along the midline. Lastly, the normal structure of the LRN is lost, the ECN is completely missing, and the IO is enlarged.

**Figure 3:**
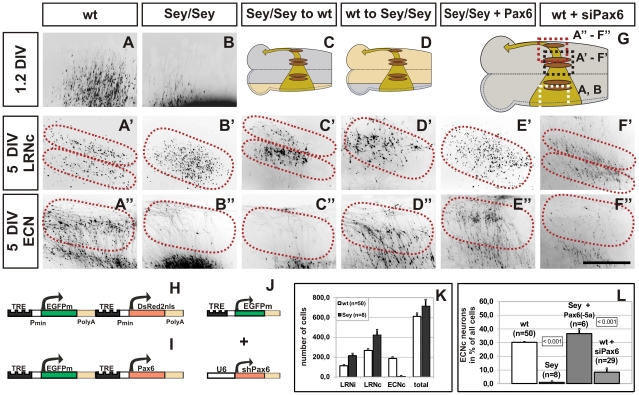
Migration defects of *Pax6^Sey/Sey^* pre-cerebellar neurons: cultures. (A–F) Fluorescence images of transfected brainstem cultures. The enlargements shown are indicated by dotted boxes in (G). Dotted lines in A′–F′ and A''–F'' indicate the LRN and ECN territories, respectively. The respective positions of the LRN and ECN are placed according to landmarks derived from wt cultures (see Figure 1). 1.2DIV wt *mms* neurons have left the rhombic lip (RL) and have almost reached the midline (A). Neurons settle after 5DIV in two sub-nuclei of the contralateral LRN (A′, LRNc) and in the ECN (A''). In contrast, *Pax6^Sey/Sey^* *mms* neurons are initially delayed (B), and then settle in a scattered LRNc (B′) but not in the ECN (B''). After transplantation of a *Pax6^Sey/Sey^* RL (beige area in C) onto a wt brainstem (grey area in C), two LRN sub-nuclei are formed (C′), but no ECN (C''), whereas, transplantation of a wt RL (grey area in D) onto a *Pax6^Sey/Sey^* brainstem (beige area in D), results in a scattered LRNc (D′) and a normal ECN (D''). Transfection of the *Pax6^Sey/Sey^* RL with *Pax6(-5a)* results in a rescue of the ECN (E''), but not of the LRNc phenotype (E′). shRNA down regulation of *Pax6* causes a loss of the ECN (F''), however, the LRNc remains unaffected (F′). Responder constructs used are: (H) for (A–D), (I) for (E), and (J) for (F), respectively. A′, B′, and E′ are red fluorescence images of the nuclear DsRednls protein; all other images are green fluorescence images of the cytoplasmic EGFP. (K) Quantification of settled LRN and ECN cells after 5DIV. There are no significant differences between the total number of migrating cells in wt and *Pax6^Sey/Sey^* cultures. (L) Quantification of neurons settling in the ECN territory after 5DIV in cultures. Standard Student's t test was used to assess the significance of changes as indicated by a p-value of <0.001; values are ±SEM. (Scale bar is 0.44 mm in [D–I''].)

In order to dissect the complex neuronal cell migration defects observed in *Pax6^Sey/Sey^* mice, *Pax6^Sey/Sey^* mice were crossed to the *Tg^(PAX6-tTA)^* transgenic line. Comparison of cultures obtained from wt and from *Pax6^Sey/Sey^* embryos confirmed the anatomical observations described above. *Pax6^Sey/Sey^* *mms* cells showed an initial delay in the onset of the migration (Figure 3A, 3B) and a disturbance at midline crossing later on. After 5 DIV (5 days in vitro) *Pax6^Sey/Sey^* *mms* neurons settled randomly in the LRN (Figure 3B′) but failed to form any ECN structures (Figure 3B''). In contrast, wt cultures formed a well organized LRN in which cells settled in a dorsal and ventral sub-nucleus of the LRN (Figure 3A′) and in a distinguished ECN (Figure 3A''). To quantify the effect we counted labeled cells in cultures from wt (LRNi  =  ipsilateral LRN  = 112±7; LRNc  =  contralateral LRN  = 266±16; ECN  =  86±14; total number of cells  = 610±34; n = 50) and *Pax6^Sey/Sey^* embryos (LRNi  = 313±23; LRNc  = 426±46; ECN  = 8±2; total number of cells  = 670±71; n = 8) (Figure 3K). These data suggested that there were no alterations in the gross number of migrating neurons between wt and *Pax6^Sey/Sey^* embryos and confirmed the complete absence of an ECN in *Pax6^Sey/Sey^* embryos. In both, mutant and wt tissues, a proportion of LRN neurons settled ipsilaterally (Figure 3K). All LRN neurons, however, projected to the contralateral cerebellum in respect to their origin from one rhombic lip, explaining the observations by Bourrat and Sotelo of an ipsilateral and contralateral contribution of mossy fibers [17].

The *Pax6^Sey/Sey^* migration defect could be caused either by a direct cell autonomous action of *Pax6* in migrating RL precursors or via an indirect non-autonomous effect, for example in the ventral domain of *Pax6*expression (e.g. by altering migration cues at the midline). We performed three types of experiments to discriminate between these alternatives. First, we transplanted transfected *Pax6^Sey/Sey^* rhombic lips onto wt brainstems and vice versa (Figure 3C, 3D). Unexpectedly, migrating *Pax6^Sey/Sey^* neurons (in a wt host) formed a well organized LRN (Figure 3C′), but no ECN (Figure 3C''). In contrast, wt neurons (in a mutant host) failed to form a correctly organized LRN (Figure 3D′), but were able to generate a normal ECN (Figure 3D''). These data suggested, that *Pax6* may act cell autonomously in generating ECN neurons, but non-autonomously in specifying the correct sub-organization of LRN neurons. To further validate this assumption we rescued the *Pax6^Sey/Sey^* migration defect by re-expression of *Pax6*. We tested the two major splice variants and of these, the expression of the *Pax6(-5a)* isoform in *Pax^Sey/Sey^*6 rhombic lips resulted in a full recovery of the ECN (Figure 3E''), but a disorganized LRN (Figure 3E′), whereas, the *Pax6(+5a*) variant was ineffective (not shown). Thus, in the RL *Pax6* splice variants differ in their biological activity, similar to the embryonic cortex [35]. Lastly, we diminished the endogenous *Pax6* mRNA by using siRNAs. Transfection of siRNAs or over expression of shRNA constructs directed against *Pax6* (Figure S4) resulted in a massive reduction of ECN cells in wt explants (Figure 3F''), whereas, control constructs had no effect (not shown). The effect was quantified by counting labeled cells that had settled in the ECN (Figure 3L). Taken together, these experiments demonstrate that our model system enables the simultaneous visualization and manipulation of tangentially migrating cells in the mouse brainstem. In addition, we have shown that *Pax6* plays numerous distinct roles in the formation and migration of mossy fiber producing neurons. Moreover, the combination of a binary model and organotypic culture assays facilitates a quick discrimination between cell-autonomous and non-autonomous effects.

### Axonal pathfinding receptors guide *mms* neurons

We identified several genes whose expression was altered in *Pax6^Sey/Sey^* *mms* neurons (Figure S5.; see also Materials and Methods). To gain more insights into the function of these putative *Pax6* downstream targets all genes were over-expressed or their expression level was diminished with shRNAs. Those genes which showed the most noticeable effects are summarized in Table 1.

**Table 1:** Summary of phenotypes obtained in organotypic cultures.

gene	Pax6^Sey/Sey^ expression	phenotype
Pax6	(↓)	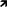 : unaltered migration
		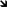 : (shRNA or siRNA or EnR) migration delay, ECN loss, midline arrest
Slit1,2	(nXII:↓)	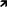 : unaltered migration
		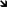 : (ablation of the Slit1-positive hypoglossal nuclei): sub-marginal migration, LRN disruption
Robo3	≈	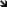 : (shRNA) sub-marginal migration, LRN disruption
Dcc	≈	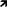 : enlarged ECN
		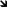 : (shRNA or siRNA) migration delay, ECN loss, altered migration path
Unc5h1	↓(RL only)	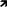 : reduced cell migration and cell death
Unc5h3	≈	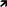 : migration delayed, altered migration path
		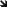 : (shRNA) unaltered migration
Neurod1	≈	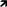 : migration delay, altered migration path
		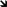 (EnR): no migration
Neurod2	≈	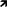 : migration delay, altered migration path
		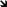 (EnR): no migration
Pou4f2	↓	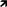 : growth cone stop at midline, altered ipsi-/contra-lateral settlement
		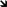 : (shRNA) midline arrest of cells
Pou4f1	↑	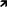 : unaltered migration
		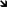 : (shRNA) unaltered migration
Math1	≈	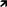 : growth cone stop at midline, altered ipsi-/contra-lateral settlement
Gap43	↓	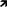 : normal migration
		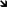 : (shRNA) altered ipsi-/contra-lateral settlement
Shh	≈	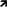 : no migration
Chordin	↓	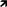 : unaltered migration
Mafb	↓	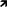 : unaltered migration

↓: gene downregulated in the Pax6^Sey/Sey^ *mms*; ≈: gene expressed similarly in the wt and the Pax6^Sey/Sey^ *mms*; ↑: gene upregulated in the Pax6^Sey/Sey^ *mms*; ?: overexpression via TRE responder constructs; 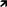 : knockdown via siRNAs or shRNA constructs, ablation of expression domain, or functional knockdown by fusion to the repressor domain of engrailed (EnR); nXII: hypoglossal nucleus;

Genes showing no or only minor mms migration phenotypes after over-expression:

Apbb2, Calpactin LC, Diras2, Gata3, Gli1, Hermes, Hip1, Internexin, Irx2, Irx6, Isl2, L1Cam, Math5, Nell2, Neurod6, Nfl, Ngn1, Ngn2, Nrp1, Olf1, Persyn, Pou6f1, RgmA, RgmB, Sema 7a, Stathmin, Syt4, Syt13.

**Figure 4:**
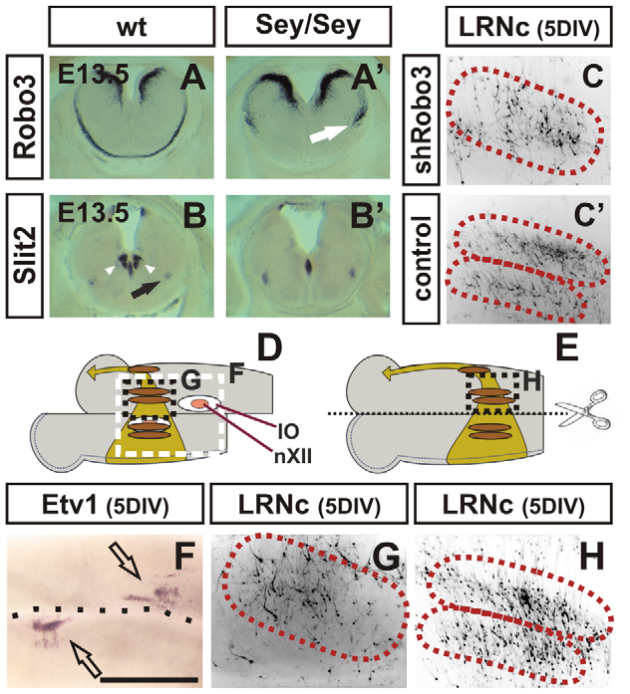
The Slit-Robo pathway controls the settlement of LRN neurons. (A,A′,B,B′) In situ hybridization of E13.5 coronal wt (A, B) and *Pax6^Sey/Sey^* (A′,B′) vibratome sections. The Slit receptor *Robo3* is expressed in neurons at the basal and alar plate and in the *mms* (A). In *Pax6^Sey/Sey^* embryos basal and alar plate expression of *Robo3* is identical to the wt expression (A′). However, *Robo3* expression in the Pax6^Sey/Sey^ *mms* again demonstrates that mutant cells are delayed in midline crossing (white arrow in A′). In wt embryos *Slit2* is expressed in a small group of cells at the midline, in the hypoglossal nuclei (arrowheads in B), in two small groups of cells at the site of the future LRN (black arrow in B), and at the ventricular zone of the rhombic lip. In *Pax6^Sey/Sey^* embryos the hypoglossal nuclei and therefore also the hypoglossal *Slit2* expression are lost, whereas, other sites of *Slit2* expression are unchanged (B′). shRNA driven knockdown of Robo3 results in a scattered LRN, suggesting that the Slit-Robo pathway controls the settling of LRN neurons (C). Control constructs had no effect on the LRNc (C′). To determine whether a local or a longitudinal distribution of guidance cues regulates the settling of LRN neurons into two sub-nuclei transplantation experiments were performed. After electroporation brainstem cultures were cut along the midline and the contralateral side was shifted to a posterior direction (D). The positions of the inferior olive (IO) and the hypoglossal nuclei (nXII) are indicated. The extent of the shift was determined by Etv1 in situ hybridization which specifically labels IO neurons (open arrows in F). (G) In a typical experiment in which the shift was larger than 500 µm neurons settled randomly in the contralateral LRN, suggesting, that cues close to the inferior olive, e.g. the hypoglossal nuclei, serve for the correct guidance of LRNc neurons. In control experiments in which there was no shift (E), LRNc neurons settled in a wt pattern and formed two groups of neurons (H). (Scale bar is 1.3 mm in [A,A′,B,B′]; 0,7 mm in [C,C′]; 0.6 mm in [F]; 0.44 mm in [G,H].)

The altered migration and settling behavior of *Pax6^Sey/Sey^* ECN/LRN neurons suggested that migration cues were changed in *Pax6^Sey/Sey^* embryos. The most prominent candidates are ligand/ receptor couples of the *Slit/ Robo*- and *Netrin/ Dcc*- pathways [36], [37]. Expression of Netrin1, Dcc, and Robo1,2 and 3 was unaltered in migrating *Pax6^Sey/Sey^* *mms* neurons (Figure 4A, 4A′, Figure S2, and data not shown). However, *Slit1* and *Slit2* which were expressed in the hypoglossal nuclei were both lost in *Pax6^Sey/Sey^* embryos (Figure 4B, 4B′ and Figure S2) [5], [10]. Motorneurons of the hypoglossal nuclei are in close proximity to the LRN settling territories suggesting that *Slit1* and *Slit2* expression provided from these neurons may determine the place of LRN settlement. To test this hypothesis we performed transplantation experiments and shRNA driven knock-down of the Slit-receptor *Robo3* in migrating *mms* neurons. Both types of experiments resulted in a disorganized LRN similar to the phenotype observed in *Pax6^Sey/Sey^* mice (Figure 4C-4H). The above results indicate that factors provided from the hypoglossal nucleus, (most likely *Slit1* and *Slit2*) determine the place of LRN settlement. These data also explain the cell non-autonomous role of *Pax6* during this process. Hypoglossal neurons are *Pax6* negative, but are completely lost in *Pax6^Sey/Sey^* embryos (Figure S2) [5], [10]; hence, *Slit1* and *Slit2* are most likely not direct targets of *Pax6*. Additional experiments suggest that *Slit1* and *Slit2* may also act as repellent to push *mms* neurons to the marginal migration route during the initial phase of migration (data not shown).

### Pou4f2 controls ipsilateral versus contralateral settling of LRN neurons and causes a migrational arrest at the midline

**Figure 5:**
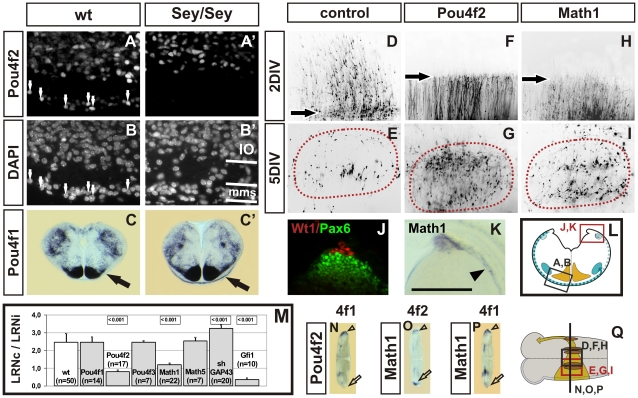
Pou4f2 induces a midline arrest of neurons in the *mms*. Immunohistochemical labeling of Pou4f2 on coronal wt (A) and Pax6^Sey/Sey^ (A′) E14.5 sections counterstained with DAPI (B and B′, respectively); high magnifications of the ventral *mms* are shown as indicated in (L). *Pou4f2* is expressed in E14.5 wt embryos in neurons of the inferior olive (IO) and in a fraction of migrating *mms* neurons (white arrows in A and B). In *Pax6^Sey/Sey^* embryos *Pou4f2* is expressed in the IO but not in neurons of the *mms* (A′,B′). In situ hybridization of coronal wt (C) and Pax6^Sey/Sey^ (C′) E15.5 sections reveals that *Pou4f1* expression is massively up-regulated in *Pax6^Sey/Sey^* embryos (arrow in C, C′). Over-expression of *Pou4f2* (F) and of *Math1* (H), but not of control constructs (D), result in a complete arrest of growth cones at the midline (black arrows in F and H) after 2DIV. In control cultures after 5DIV few cells settle in the ipsilateral LRN (LRNi) (E), whereas, in cultures over-expressing *Pou4f2* or *Math1* a significant higher proportion of cells settles in the LRNi (G, I). (J, K) Distribution of Wt1, Pax6 and *Math1* on coronal wt E14.5 sections as indicated in (L). (J) Immunohistochemical labeling of Pax6 (green) and Wt1 (red) indicates that Wt1 is expressed in the roof plate dorsally to the rhombic lip and that Pax6 and Wt1 are non-overlapping. (K) In situ hybridization with *Math1* indicates that *Math1* is expressed at the rhombic lip and in a subset of cells emanating from the rhombic lip (arrowhead in K). (M) Quantification of *mms* neurons settling in the LRN; values are given for the proportion of cells settling in the LRNc or LRNi. The significance of changes is indicated by a p-value of <0.001; values are ±SEM. Over-expression of *Pou4f2, Math1*, and *Gfi1* resulted in a higher ratio of LRNi cells and down-regulation of *Gap43* led to a lower ratio of LRNi cells. (N-P) In situ hybridization of sectioned cultures after over-expression of *Pou4f2* or *Math1. Pou4f1* expression is reduced in *Pou4f2* and in *Math1* transfected rhombic lips (open arrows in N,P), but not in the control rhombic lip (open arrowheads in N,P). *Pou4f2* is up-regulated in *Math1* transfected (open arrow in O) but not in control (open arrowhead in O) rhombic lips. (Scale bar is 110 µm in [A, A′, B, B′, J, K]; 1,4mm in [C, C′, N, O, P].); 440 µm in [D–I].)

Two POU transcription factors were among the genes whose expression pattern was altered in the *mms* of *Pax6^Sey/Sey^* embryos. Pou4f2 (also: *Brn3b*) was strongly expressed in about 18.6% (±4.6%, n = 3) of E14.5 and 23.3% (±6.2%, n = 3) of E15.5 wt *mms* neurons but was completely lost in the *Pax6^Sey/Sey^* *mms* (Figure 5A, 5A′, 5B, 5B′). *Pou4f1* (also: Brn3a) was expressed between E13.5 and E15.5 in a subset of *mms* neurons, but was up-regulated in the E14.5 and E15.5 *Pax6^Sey/Sey^* *mms* (Figure 5C, 5C′). Expression of *Pou4f1* and *Pou4f2* in *Pax6^Sey/Sey^* IO neurons was unaltered (Figure 5A, 5A′, 5C, 5C′). *Pou4f2* plays several roles in specifying and guiding retinal ganglion cells and their axons. We therefore asked whether *Pou4f2* may accomplish similar tasks in rhombic lip derived neurons. *Pou4f2* was only expressed in a subset of wt *mms* neurons. We therefore over-expressed *Pou4f2* in all migrating *mms* neurons. Remarkably, growth cones of all *Pou4f2* over-expressing neurons were arrested at the midline for about 1.5 days (±0.5 days, n = 17), whereas the majority axons in control cultures crossed the midline instantly (Figure 5D, 5F). Interestingly, in control cultures the growth cones of some neurons also appeared to be arrested at the midline: 5% (±3%) at 1DIV, 15% (±6%) at 2DIV, 25% (±5%) at 3DIV, and 6% (±3%) at 4DIV (n = 11). This correlates well to the peak of Pou4f2 expression at E14.4 and E15.5 (in cultures: 2DIV and 3DIV). Over-expression of *Pou4f2* had also a noticeable effect on the settling behavior of LRN neurons. Quantification of LRN neurons at 5DIV revealed that *Pou4f2* expressing LRN neurons preferably settled at the ispilateral side (LRNc/LRNi  = 0.8 ± 0.1, n = 17; Figure 5G, 5M) compared to control cultures in which the majority of LRN neurons settled at the contralateral side (LRNc/LRNi  = 2.5 ±0.1, n = 50; Figure 5E, 5M). Similar relations were obtained at 6DIV and 8DIV suggesting that *Pou4f2* over-expression altered the migration behavior of *mms* neurons and did not cause a delayed settlement of these neurons. The effect was specific to *Pou4f2* and could not be mimicked by over-expression of *Pou4f1*, *Pou4f3* or *Pou6f1* (Figure 5M and data not shown). Together these data suggest that *Pou4f2* acts through a novel mechanism which induces an arrest of growth cones at the midline to regulate the ratio of ipsilaterally versus contralaterally settling neurons.

We altered expression levels of about 25 potential *Pou4f2* retinal target genes [38]–[40] and of these two showed an effect on the migration behavior of *mms* neurons. Over-expression of *Gfi1*, a zinc finger transcription factor, reduced the contra-/ipsi-lateral ratio of LRN neurons (Figure 5M). In contrast, the down-regulation of *Gap43* by shRNA constructs caused a higher contra-/ipsi-lateral ratio of LRN neurons (Figure 5M and Figure S4). *Gap43* is slightly reduced in the *Pax6^Sey/Sey^* *mms* (Figure S5). In addition, mis-expression of *Pou4f2* resulted in a massive down-regulation of *Pou4f1* in transfected, but not in control, rhombic lips (Figure 5N), suggesting that the loss of *Pou4f2* in Pax6^Sey/Sey^ *mms* neurons leads to an up-regulation of *Pou4f1* (Figure 5C, 5C′). *Pou4f1* over-expression or down-regulation, however, did not alter migration behavior of *mms* neurons (Figure 5M).

*Pou4f2* is expressed only in a subset of *Pax6* positive *mms* neurons suggesting that other factors together with *Pax6* may co-regulate *Pou4f2*. In the developing retina *Pou4f2* expression depends on two transcription factors: the bHLH protein *Math5* and the zinc finger gene *Wt1* [41]–[44]. *Wt1* was found to be expressed in the rhombic lip, though, in a region just dorsally to the *Pax6* positive domain (Figure 5J). *Math5* was neither expressed in the rhombic lip nor in migrating *mms* neurons, however, a close homologue, *Math1*, was expressed in neuronal precursors at the rhombic lip and in a subset of early migrating *mms* neurons [22], [23] (Figure 5K). Thus, *Math1*, but neither *Math5* nor *Wt1*, was the most likely candidate to regulate *Pou4f2* or *Pou4f1* expression in *mms* neurons. Consistent with this, mis-expression of *Math1*, but not of *Wt1* (+ and – KTS splice variants) or *Math5*, led to a midline arrest of migrating *mms* neurons and a reversed settling behavior of LRN neurons (Figure 5H, 5I, 5M). Mis-expression of *Math1* resulted in an up-regulation of *Pou4f2* and a down-regulation of *Pou4f1* (Figure 5O, 5P). Together, these data suggest, that *Pou4f2* expression in rhombic lip derived *mms* neurons depends on *Pax6* and *Math1* and that *Pou4f2* may regulate *Pou4f1* and *Gap43* in *mms* neurons.

In summary, our work led to the identification of a gene cascade acting in tangentially migrating neurons of the brainstem, in which *Pou4f2* plays a central role to induce a previously unknown mechanism that controls midline crossing behavior. Furthermore, our results imply that our model system is applicable to quickly analyze genetic hierarchies in *Pax6* positive cells and may therefore serve as a general tool.

## Discussion

### *Tg^(PAX6-tTA)^* mice as a model for Pax6 function, cell migration, and axonal pathfinding processes

The extraordinary complexity of cell determination, migration and wiring processes in the developing mammalian brain creates a major challenge for developmental neurobiologists. Here, we introduced a simple yet powerful technology to quickly analyze any gene potentially involved in these processes. Our model is of threefold use: first to study the function of *Pax6* and of *Pax6* downstream genes in their genuine environment, second to investigate genes involved in general patterning, axonal pathfinding and cell migration processes, and third to enable the analysis of tissue-specific gene functions. The *Tg^(PAX6-tTA)^* model complements and improves existing approaches and has certain benefits: it combines cell specific transfection protocols and organotypic culture assays, thus, facilitating the quick analysis of genes in a natural tissue environment. The experimental design and the binary nature of the *Tg^(PAX6-tTA)^* model is fundamentally simple and has several advantages over systems that are based purely on transgenic animals. First, the electroporation and subsequent culture of embryonic tissues allows the screening of large number of genes without the need of generating new transgenic animals for each construct. In fact, less than 10% of the constructs we have tested revealed phenotypes. Thus, only those genes showing positive results in culture assays may be used subsequently to generate transgenic lines. Of note however, some of the phenotypes reported here, for example, the midline arrest or the altered ipsi- to contra-lateral ratio, would have been missed in purely transgenic systems. Second, variation of the electroporation protocol allows transfections ranging from just a few cells to a complete *Pax6* expression domain with thousands of cells. Hence, our approach allows adjustment according to the needs: either to monitor single migrating cells or to determine global patterning effects. In addition, neighboring cells and non-electroporated contra-lateral sides serve as internal controls. The usefulness of our system critically depends on the tightness of the TRE based promoter and on the ability of the constructs to express two genes simultaneously. To ascertain the tightness of our system we used repeated electroporations and high DNA concentrations (up to 5 µg/µl). Even under these extreme conditions we were never able to detect any reporter gene expression in *Pax6* negative cells at any developmental stage. Thus, under the conditions used in this report the combination of *Tg^(PAX6-tTA)^* mice and TRE based promoters allow expression of reporter gene constructs only in *Pax6* positive cells. It is also important to note, that our strategy to use a YAC based technology combined with an internal ribosomal entry site (IRES) resulted in moderate levels of reporter gene expression which were in the range of physiological concentrations. To ensure the simultaneous expression of two reporter genes we tested several types of TRE constructs. Only our approach, to use two consecutive TRE based promoters led to the activation of nearly equal amounts of two genes at the same time in the same cell. A bidirectional TRE element that previously had been shown to work in transgenic animals failed in our system [45]. One obvious difference is that in transgenic animals typically multiple copies of constructs are stably integrated into the genome, whereas, in our assay transfections were transient.

*Pax6* loss of function phenotypes are often highly complex involving massive malformations in the affected organs. *Pax6* is expressed in neuronal precursors of the telencephalon, commissural neurons in the dorsal spinal cord, in adult neuronal stem cells, the early eye cup, in the pancreas, in precursors and in migrating cells of several tangential and radial migration streams of the rhombencephalon and of the forebrain [5], [6], [10], [11], [14], [28], [46], [47]. In addition to its technical advances, the *Tg^(PAX6-tTA)^* model represents a novel, highly versatile technology to study the function of *Pax6* or any other gene in these tissues. As a paradigm, we have dissected the role of *Pax6* in tangentially migrating cells of the brainstem. In principle, however, this system shall be applicable to any *Pax6* positive tissue and we have initial evidence that our model allows to specifically target *Pax6* positive telencephalic precursor cells, cerebellar granule cells, the developing retina, the rostral migratory stream, the pontine migration and ventral precursor cells of the brainstem and spinal cord (Figure S1 and data not shown). With the help of this model it should therefore be possible to systematically analyze cell fate decisions and the migratory behavior of *Pax6* expressing cells at any developmental stage.

**Figure 6:**
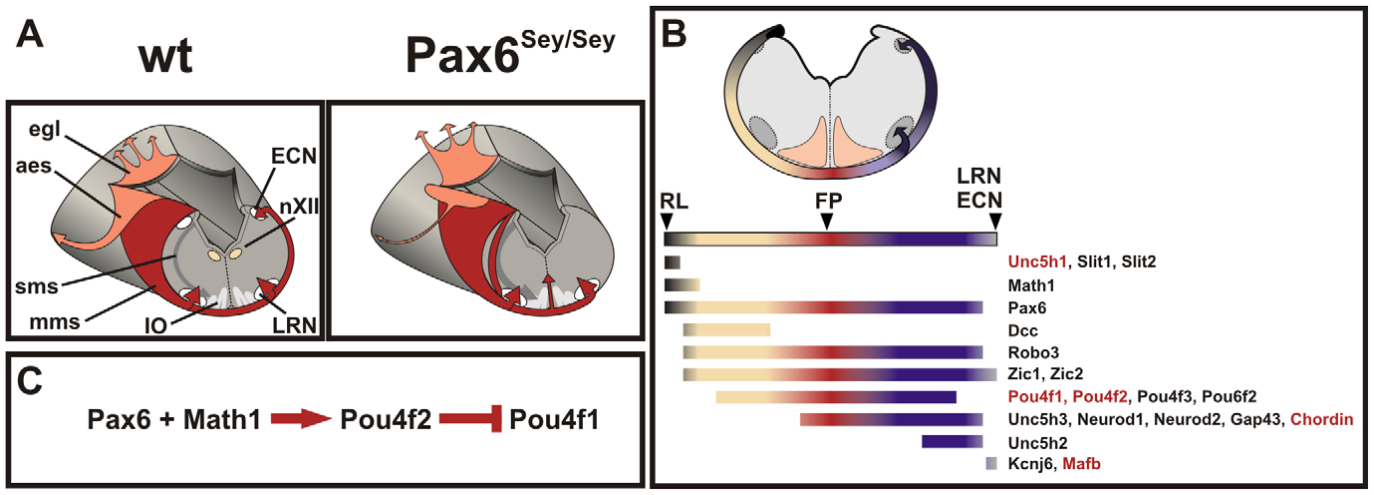
Summary of phenotypes observed in *Pax6^Sey/Sey^* RL derived neurons. (A) In *Pax6^Sey/Sey^* embryos the *mms* migration is delayed, some neurons migrate sub-marginally, neurons are arrested at the midline, the ECN is lost, the LRN is disrupted, and the IO is enlarged [16]. Additionally, the *aes* is reduced resulting in an absence of the pontine nuclei and granule cells of the cerebellum migrate ectopically into the inferior culliculus [14]. To search for genes expressed in *mms* neurons a large scale in situ hybridization screen was performed and a summary of the expression data of selected genes is shown in (B). The color code indicates expression of individual genes during migration of *mms* neurons: dark grey marks expression at the RL, beige before midline crossing, red during midline crossing, blue after crossing the midline and light grey marks expression during settlement in the ECN and LRN. Genes, which show altered expression levels in *Pax6^Sey/Sey^* embryos are marked in red. (C) A gene cascade in which *Pax6* and *Math1* positively regulate *Pou4f2* expression leads to a repression of *Pou4f1* expression. *mms*: marginal migration stream forming the external cuneate (ECN) and lateral reticular (LRN) nuclei; *sms*: sub-marginal migration stream forming the inferior olive (IO); *aes*: anterior extramural migration stream forming the pontine and pontine reticulotegmental nuclei; *egl*: external granule cell layer of the cerebellum; RL: rhombic lip; FP: floor plate.

Several studies have revealed that *Pax6* is required for hindbrain and spinal cord development [5], [7], [10], [14], [15]. Our work adds that *Pax6* also controls the determination and migration of rhombic lip derived neurons (for a summary see Table 1 and Figure 6). *Pax6* functions twofold: first, *Pax6* controls guidance cues which push migrating *mms* neurons to the marginal path and which control the settling pattern of LRN neurons. The most likely sources of these cues are the hypoglossal nuclei which are located close to the midline and in proximity to the LRN. *Slit1* and *Slit2* are expressed in the hypoglossal nuclei and the *Slit* receptor *Robo3* is expressed in migrating *mms* neurons [48]. *Slit* expression provided by the hypoglossal nuclei may therefore act as repellent to push *mms* neurons to a marginal migration route and may also specify the settlement of neurons in the LRN. The loss of *Slit*-expressing hypoglossal nuclei in *Pax6^Sey/Sey^* embryos [5], [10] causes a major reduction of the repellent (a minor source of *Slit* is still present in midline cells). Consequently, migrating *mms* neurons would use a more sub-marginal migration route and settle less organized in *Pax6^Sey/Sey^* embryos. Furthermore, *Slit* expression at the RL may be involved during the initial phase of *mms* migration. Secondly, *Pax6* functions cell-autonomously in migrating *mms* neurons to control the determination, the timing of migration, and midline crossing. Several genes show altered expression in *Pax6^Sey/Sey^* *mms* neurons (Table 1: *Pou4f1*, *Pou4f2*, *Unc5h1*, *Mafb*, Chordin) and may convey individual aspects of migration.

We and others find that several transcription factors relay *Pax6* downstream effects in dorsal brainstem neurons: *Ngn1* in precursor cells ventral to the RL [16], and *Pou4f1*, and *Pou4f2* in migrating neurons (this report). Mis-expression of *Ngn1* or *Ngn2* in *Pax6^Sey/Sey^* embryos failed to rescue the migration defects observed in the *Pax6* mutant (Table 1). Neither did the mis-expression or down-regulation of these genes generate *small eye* - like migration defects in wt embryos (Table 1). On the other hand, *Pou4f2*, which is lost in the *mms* of *Pax6^Sey/Sey^* embryos (Figure 5B and also in the pontine migration and in the cerebellum, data not shown), alters migration behavior of *mms* neurons. Together these data suggest that *Pou4f2* may regulate genes involved in pathfinding processes, whereas, *Ngn1* acts earlier in the cell determination process.

There are striking similarities in gene expression pattern between sensory neurons and RL derived neurons. We found that at least two thirds of the genes which are co-expressed with *Pax6* and *Pou4f2* in retinal ganglion cells are also co-expressed with these genes in *mms* neurons. Furthermore, genetic hierarchies seem to be analogous: in the retina *Math5* controls *Pou4f2*, which then acts upstream of *Pou4f1* [38], [41]–[43], whereas, in RL derived neurons *Math1*, a close homologue of *Math5*, initiates related pathways. General genetic pathways are conserved between retinal and RL derived neurons and our model may therefore help to elucidate some of the phenotypes observed in *Pou4f1^-/-^* and *Pou4f2^-/-^* mice. Both mouse models have revealed distinct axonal pathfinding errors [39], [49]–[52]. Mis-expression of *Pou4f2* (or *Math1*) in RL derived neurons stalls growth cones at the midline for several hours. To our knowledge, this is the first report of such a midline arrest and it may thereby be a paradigm for a novel mechanism controlling midline crossing. The arrest does neither induce a growth cone collapse nor does it inhibit midline crossing per se as all neurons generate axons that cross the midline after a “waiting period”. These axons all migrated into the cerebellum like those of control cultures. *Gfi1* mis-expression and *Gap43* knockdown were able to partially mimic the *Pou4f2* induced phenotype, however, additional unknown targets or a combinatory code may be needed to elicit the full phenotype.

As *Pou4f2* was only expressed in about 1/4 of wt *mms* neurons, the loss of *Pou4f2* in *Pax6^Sey/Sey^* embryos mimics only minor aspects of the *Pax6^Sey/Sey^* phenotype. The down regulation of *Pou4f2* by shRNA constructs resulted in a severe midline disturbance of neuronal processes at similar to the phenotype observed in *Pax6^Sey/Sey^* cultures, whereas, in control cultures neuronal processes crossed the midline instantly (data not shown). Comparable phenotypes were also observed in *Pax6^Sey/Sey^* cultures, in cultures transfected with *Pax6* shRNA constructs, and in *Pax6^Sey/Sey^* brainstem sections.

Tangentially migrating neurons follow similar navigational cues as developing axons [14], [19], [48], [53]–[58]. Hence, tangentially migrating neurons of the *mms* provide an excellent system to study axonal pathfinding and neuronal cell migration processes. Migrating *mms* neurons are easily accessible as they navigate along the pial surface. Our model takes advantage of the superficial migration of these neurons and provides a straightforward assay to specifically label and manipulate these cells without affecting their surroundings. Members of most families of guidance receptors (N*etrin* receptors, *Slit* receptors, *Semaphorins*, *Eph* receptors, and *Ephrins*) are expressed in migrating *mms* neurons [48], [53], [54] (Engelkamp, unpublished) and at least two of these pathways are essential for the correct guidance of *mms* neurons: the Slit/ Robo *-* [48] and the Netrin/ Dcc*-* pathways [53], [56], [57] (see also Table 1). Our system should therefore also have important implications for the study of the signal cascades entailed in these pathways.

In summary, we have established a novel model system which allows the simultaneous visualization and manipulation of neuronal subpopulations. As a prototypical model we have focused on the role of *Pax6* in migrating brainstem neurons. Yet, our results imply that our model system is applicable to a range of other cells in the developing brain and may therefore serve as a general tool to quickly study axonal pathfinding, neuronal cell migration or patterning processes.

## Materials and Methods

### Animals

The *Small Eye* allele [4] was maintained on a CD1 background. Embryos were obtained from matings of heterozygote (*Pax6^Sey/+^*) mice. 0.5 denotes the morning when the vaginal plug was found. Experiments were always performed on matching pairs of control (wt) and *Pax6^Sey/Sey^* embryos that were carefully staged. All phenotypes described were confirmed on at least six individual *Pax6^Sey/Sey^* embryos obtained from different crossings. There was no noticeable phenotypic difference between *Pax6^Sey/+^* and wt embryos and therefore, in our experiments wt designates wt and *Pax6^Sey/+^* embryos. For brainstem cultures, matings between heterozygote *Tg^(PAX6-tTA)^* and wt CD1 mice or between heterozygote *Pax6^Sey/+^*/ *Tg^(PAX6-tTA)^* and heterozygote *Pax6^Sey/+^* mice (to generate *Pax6^Sey/Sey^* cultures) were used. Genotyping was performed by PCR with primers directed against the Tet repressor (upper: GCGCTGTGGGGCATTTTACTTTAG; lower: CCGCCAGCCCCGCCTCTTC). All animal procedures were carried out in accordance to the guideline approved by institutional protocols.

### Generation of *Tg^(PAX6-tTA)^* mice

YAC *Y593* [30] was modified such that exons 8 to 11 of the *Pax6* gene were replaced by homologous recombination with a construct containing the following elements in 5′ to 3′ order: *Pax6k30 – IRES – tTA – loxP – LYS2 – loxP – Pax6k32*. *Pax6k30* and *Pax6k32* corresponded to the sequences 29.792 to 30.296 and 31.587 to 32.095 of the *Pax6* cosmid *cFAT5* (NCBI accession no. Z95332), respectively, and were generated via PCR. The *IRES* (internal ribosomal entry site) was derived from *pIRES-EGFP* (Invitrogen), however, the original ATG-11 start codon was reconstituted to enhance translational initiation. tTA (Tet-On-system), a fusion of the tetracycline repressor and the activation domain of VP16, was derived from pUHD15-1neo (Clontech). The *LYS2* gene from S. cerevisiae was derived from *pAF107*, which was obtained from B. Dujon, Institute Pasteur, Paris, France [59]. *LoxP* sequences were generated via PCR. All constructs were sequence verified. Homologous recombination in yeast was performed using standard techniques. The integrity of the recombined YAC was then verified by PCR and southern blotting. Preparation of the YAC DNA and the generation of transgenic mice were as described [30].

### In situ hybridisation

In situ hybridization was performed on free floating vibratome sections as previously described [14]. Probes for *Math1* [60], *Neurod1* and *Neurod2* [61], *Pax6* [47], *Unc5h3* [62] and *Slit1*, *Slit2* [63] were obtained from H. Zoghbi, A. Bartholomä, R. Hill, S. Ackerman, and M. Little, respectively. Probes for *Dcc* and *RgmB* were as published [34], [64]; other probes were obtained by RT-PCR. The PCR products were subcloned and their identities were confirmed by sequencing. The general staining patterns of all probes matched published expression patterns. Probes were as follows: *Etv1* (bp 853–1820 of NM_007960); *Fgfr2* (bp 343–1192 of NM_201601); *Pou4f1* (bp 1321–2199 of NM_011143), *Pou4f2* (bp 216 – 1762 of S68377); *Robo3* (bp 3648–4673 of NM_011248). Several genes which are down- or up-regulated in Pax6^Sey/Sey^ embryos were identified with the help of a large scale in situ screen using >300 putative candidates. Individual probes are available on request.

### Recombinant constructs

The vector for the co-expression of two constructs in *Tg^(PAX6-tTA)^* mice contained the following elements in 5′ to 3′ order: MCSI – TRE – P_min_CMV – IntronA - BGHPolyA – MCSII – TRE - SV40PolyA; MCS  =  multiple cloning sites; TRE  = 7 repeats of the tetracycline responsive element, P_min_CMV  =  minimal CMV promoter, and IntronA were from ptetOi-MCS (obtained from Martin Spiegel, Tübingen); SV40polyA and BGHPolyA  =  polyadenylation signals (derived from pTetOi-MCS and pRc/CMV, Invitrogen, respectively). Fluorescent markers to label migrating cells were a modified EGFP or DsRed2 (Clontech). Full length clones for *Gfi1, Math1, Math5, Ngn1*, and *Ngn2* were obtained from the German Resource Center for Genome Research (RZPD) and sequence verified; clones for all other genes were obtained by RT-PCR and confirmed by sequencing. Fusions with a triple HA-tag or the engrailed repressor domain (EnR) were generated by PCR. shRNA constructs were generated in the psiSTRIKE vector (Promega) using the Promega Web tool for designing the hairpin oligonucleotides. In the psiSTRIKE vector shRNAs are expressed under control of the U6 RNA polymerase promoter. Efficiency of shRNA knockdown was demonstrated in HEK293 cells using the psiCHECK/ Dual Luciferase system according to the manufacturers protocol (Promega). All constructs were sequence verified.

### Brainstem cultures

Responder constructs (2-4 µl at 0.5 µg/µl in GBSS/ 0.01% Methyl Fast, Sigma) were injected into the fourth ventricle of E12.5 wt and *Tg^(PAX6-tTA)^* mouse embryos by using glass needles. Electroporation was then performed with forceps-like electrodes with platinum ending (Ø = 0.5 mm) (one Electrode above the right RL and the other under the left jaw). Conditions were 8 pulses at 50V, 50msec with a pulse interval of 1sec. We used the square pulse generator EPI2500 (L. Fischer, Heidelberg). After electroporation, the hindbrain (rhombomeres 1–8 including the cerebellar anlage) was dissected, opened at the roof plate and cultivated with the ventricular site onto MillicellCM filters (Millipore) in culture medium (DMEM/F12 (1∶1); 0.6% Glucose; 0.02 mM Glutamine; 5 mM HEPES; 5% Fetal Calf Serum; 5% Horse Serum) at 37°C and 5%CO_2_.

### Immunohistochemistry

Depending on the antibodies used, brainstem preparations were fixed with 4% or 0.2% PFA in PBS for 12 hours at 4°C. Cryosections were cut at 14 µm. Primary and secondary antibodies used for staining were as follows: mouse monoclonal (mAb) α-Pax6 ([10], 1∶1000, DSHB); rabbit pAb α-Pou4f2 (also Brn3b, 1∶300, Covance); rabbit pAb α-Kcnj6 (also Girk2, 1∶300, Chemicon); rabbit α-Wt1 (Santa Cruz); rabbit α-VP16 (Clontech); α-HA-tag (1∶100, Roche) and α-mouse and α-rabbit secondary antibodies conjugated with Alexa488 or Alexa596 (Molecular Probes). Quantification of Pou4f2 positive *mms* neurons was done on every 3^rd^ of serial sections double stained for Pax6 and Pou4f2.

### Image analysis

Images were taken at a Zeiss Axiophot microscope equipped with a Spot camera, at a confocal Zeiss LSM microscope, or at a Leica MZ12 equipped with a camera device. Images were processed using the MetaView software (Universal Imaging Corporation) and Adobe Photoshop. To perform statistical analysis the position of the ECN and the LRN were determined in wt un-manipulated cultures by in situ RNA staining of *Pax6* and *Kcnj6*. The resulting territories were then overlaid onto the electroporated cultures with the help of three landmarks: a) the position of the rhombic lip; b) the position of the floor plate; and c) the position of the superior and inferior olivary complexes, which both are visible in phase contrast images of the cultures. This procedure allowed classifying 97% of labeled neurons on the contralateral side and 90% on the ipsilateral side as either ECN or LRN neurons. The remaining 3% (or 10% for the ipsilateral side) of labeled cells were scattered neurons mainly in between the ECN and the LRN. Quantification of growth cones arrested at the midline in wt cultures was done by counting all growth cones in a 25 µm wide territory at the midline. Continuous observations of cultures implied that *mms* growth cones traveled at an average speed of at least 500 µm/day, suggesting that within any 25 µm interval only 5% of growth cones should be detected if migration would not pause. Quantification of the volume of the inferior olive was done with AxioVision (Zeiss).

## Supporting Information

Figure S1 Pax6, tTA and EGFP expression in tg^(PAX6-tTA)^ embryos. (A,B,E,F,I,J) In situ hybridizations of coronal vibratome sections of tg^(PAX6-tTA)^ transgenic embryos. Alternating sections were stained for either *Pax6* (A,E,I) or tTA (B,F,J). In the developing eye, *Pax6* and tTA are co-expressed in the neural retina (nr), the lens, and the surface ectoderm (s.e.) which generates the future cornea. In the forebrain, both genes are co-expressed in the cortex (ctx), the ventral diencephalon (di), and the epithalamus (epi). In the cerebellum, *Pax6* and tTA are co-expressed in granule cells of the external granule cell layer (egl). (C,D,G,H,K,L) Electroporation of EGFP reporter constructs into tg^(PAX6-tTA)^ transgenic embryos. After electroporation tissues were cultured for one or two days on MilliporeCM filters. Reporter gene expression is activated in the developing retina, but not in the surrounding tissue (C); (D) Higher magnification of retinal precursor cells. (G) Electroporation of the telencephalon at E13.5 results in a specific labeling of the *Pax6* positive cortex (ctx, blue dotted line), but not of the *Pax6* negative striatum (red dots) or other surrounding tissues. (H) Electroporation of the E18.5 anterior telencephalon reveals precursor cells with the typical appearance of radial glial cells; vz, svz, iz and cp denote the ventricular, subventricular, and intermediate zones, and the cortical plate, respectively. (K) In the developing cerebellum EGFP expression is seen in granule cells which leave the transfected region and start to migrate parallel to the cerebellar surface. (L) Higher magnification of (K). Scale bar: 0.6 mm in [A,B]; 1.9 mm in [E,F]; 0.8 mm in [I,J]; 400 µm in [C,H, K]; 1.7 mm in [G]; and 150 µm in [D,L].
(TIF)

Figure S2 *Pax6^Sey/Sey^* Brainstem defects. The hypoglossal nucleus (black arrows) is lost in *Pax6^Sey/Sey^* embryos as shown by *Slit2*, *Isl1*, *L1Cam*, and *Unc5h3* labelling (A–D). Migrating mms neurons (white arrows) take a submarginal route in *Pax6^Sey/Sey^* embryos as shown by *Robo3*, *Dcc*, and *Cntn2* (also: *Tag1*) staining (E–I). *Zic1* marks *mms* neurons which ectopically settle in the IO territory of *Pax6^Sey/Sey^* embryos (J). Red arrows indicate positions of the ECN and LRN and red arrowheads indicate ectopic settlement of *mms* neurons in *Pax6^Sey/Sey^* embryos. (Scale bar is 0.3mm in [A, A′]; 0.2 mm in [B, B′, C, C′, D, D′, F, F′, H, H′]; 0.9 mm in [E, E′]; 1 mm in [G, G′]; 1.2 mm in [I, I′]; 2 mm in [J, J′].)
(TIF)

Figure S3 Increased size of the *Pax6^Sey/Sey^* inferior olive. (A) Complete series of coronal E14.5 wt and *Pax6^Sey/Sey^* brainstem vibratome sections stained with *Etv1* by in situ hybridization. (B) Examples of the size determination of the inferior olivary nuclei. Shown are two individual pairs of E14.5 embryos stained with *Etv1* and one pair of E14.5 embryos stained with *Pou4f2*. Note that *Etv1* is a specific marker for inferior olivary cells, whereas, *Pou4f2* stains *mms* and inferior olivary neurons and may therefore also include *mms* neurons that have ectopically migrated into the inferior olivary territory. Values are given in µm^2^ for the area taken by the left plus right inferior olivary sub-nuclei. (C,D,E,F) In situ hybridization of coronal wt (C,D,E) and *Pax6^Sey/Sey^* (F) E14.5 (C,D) and E18.5 (E,F) vibratome sections. *Fgfr2a* (C) and *Unc5h3* (D) label both: migrating *mms* neurons and IO neurons. *RgmB* labels equally the dorsal and principal sub-nuclei of the wt and the *Pax6^Sey/Sey^* inferior olive suggesting a normal patterning of the mutant inferior olivary nucleus. Scale bar: 0.44 mm in [A]; 0.7 mm in [C,D]; 0.55 mm in [E,F].
(TIF)

Figure S4 Efficiency of shRNA and siRNA gene knockdown. (A–D) Efficiency of RNA knockdown. The fold repression was determined by the degree of silencing of Renilla luciferase-targeting construct relative to the firefly luciferase control. Averages of two to four individual experiments are shown as indicated. (E) The target sequences of shRNA constructs as indicated by their positions in *Pax6* (accession # NM_123627), *Robo3* (AF060570), *Pou4f2* (S68377), and *Gap43* (NM_008083) cDNAs. Dharmacon siRNA SMART pools consist of a pool of 3 individual siRNAs; the sequence is not provided.
(TIF)

Figure S5 Altered gene expression in the *Pax6^Sey/Sey^* hindbrain. Differential expression of putative Pax6 downstream genes in the RL (A,B), the *mms* (C–H) and the cerebellum (I–K). The dotted line in B, B′ demarcates the boundary between the rhombic lip (open arrowhead) and the remaining alar plate. White arrows in I–K indicate the external granule cell layer (egl). (Scale bar is 0.4 mm in [A, A′]; 0.2 mm in [B, B′]; 0.7 mm in [C, C′, D, D′, E, E′, F, F′, I, I′, J, J′, K, K′]; 0.3 mm in [G, G′, H, H′].)
(TIF)

## References

[1] van Heyningen V, Williamson KA (2002). PAX6 in sensory development.. Human Molecular Genetics.

[2] Pichaud F, Desplan C (2002). Pax genes and eye organogenesis.. Current Opinion in Genetics & Development.

[3] Simpson TI, Price DJ (2002). Pax6; a pleiotropic player in development.. Bioessays.

[4] Hill RE, Favor J, Hogan BL, Ton CC, Saunders GF (1991). Mouse small eye results from mutations in a paired-like homeobox-containing gene [published erratum appears in Nature 1992 Feb 20;355(6362):750].. Nature.

[5] Osumi N, Hirota A, Ohuchi H, Nakafuku M, Iimura T (1997). Pax-6 is involved in the specification of hindbrain motor neuron subtype.. Development.

[6] Stoykova A, Fritsch R, Walther C, Gruss P (1996). Forebrain patterning defects in Small eye mutant mice.. Development.

[7] Takahashi M, Osumi N (2002). Pax6 regulates specification of ventral neurone subtypes in the hindbrain by establishing progenitor domains.. Development.

[8] Warren N, Caric D, Pratt T, Clausen JA, Asavaritikrai P (1999). The transcription factor, Pax6, is required for cell proliferation and differentiation in the developing cerebral cortex.. Cerebral Cortex.

[9] Vitalis T, Cases O, Engelkamp D, Verney C, Price DJ (2000). Defects of tyrosine hydroxylase-immunoreactive neurons in the brains of mice lacking the transcription factor Pax6.. Journal of Neuroscience.

[10] Ericson J, Rashbass P, Schedl A, Brenner-Morton S, Kawakami A (1997). Pax6 controls progenitor cell identity and neuronal fate in response to graded Shh signaling.. Cell.

[11] Heins N, Malatesta P, Cecconi F, Nakafuku M, Tucker KL (2002). Glial cells generate neurons: the role of the transcription factor Pax6.. Nature Neuroscience.

[12] Götz M, Stoykova A, Gruss P (1998). Pax6 controls radial glia differentiation in the cerebral cortex.. Neuron.

[13] Estivill-Torrús G, Vitalis T, Fernández-Llebrez P, Price DJ (2001). The transcription factor Pax6 is required for development of the diencephalic dorsal midline secretory radial glia that form the subcommissural organ.. Mechanisms of Development.

[14] Engelkamp D, Rashbass P, Seawright A, van Heyningen V (1999). Role of Pax6 in development of the cerebellar system.. Development.

[15] Yamasaki T, Kawaji K, Ono K, Bito H, Hirano T (2001). Pax6 regulates granule cell polarization during parallel fiber formation in the developing cerebellum.. Development.

[16] Landsberg RL, Awatramani RB, Hunter NL, Farago AF, DiPietrantonio HJ (2005). Hindbrain rhombic lip is comprised of discrete progenitor cell populations allocated by Pax6.. Neuron.

[17] Bourrat F, Sotelo C (1990). Migratory pathways and selective aggregation of the lateral reticular neurons in the rat embryo: a horseradish peroxidase in vitro study, with special reference to migration patterns of the precerebellar nuclei.. Journal of Comparative Neurology.

[18] Rodriguez CI, Dymecki SM (2000). Origin of the precerebellar system.. Neuron.

[19] Yee KT, Simon HH, Tessier-Lavigne M, O'Leary DDM (1999). Extension of long leading processes and neuronal migration in the mammalian brain directed by the chemoattractant netrin-1.. Neuron.

[20] Wingate RJT (2001). The rhombic lip and early cerebellar development.. Current Opinion in Neurobiology.

[21] Kyriakopoulou K, de Diego I, Wassef M, Karagogeos D (2002). A combination of chain and neurophilic migration involving the adhesion molecule TAG-1 in the caudal medulla.. Development.

[22] Machold R, Fishell G (2005). Math1 is expressed in temporally discrete pools of cerebellar rhombic-lip neural progenitors.. Neuron.

[23] Wang VY, Rose MF, Zoghbi HY (2005). Math1 Expression Redefines the Rhombic Lip Derivatives and Reveals Novel Lineages within the Brainstem and Cerebellum.. Neuron.

[24] Ray RS, Dymecki SM (2009). Rautenlippe Redux -- toward a unified view of the precerebellar rhombic lip.. Curr Opin Cell Biol.

[25] Altman J, Bayer SA (1987). Development of the precerebellar nuclei in the rat: III. The posterior precerebellar extramural migratory stream and the lateral reticular and external cuneate nuclei.. Journal of Comparative Neurology.

[26] Dickson BJ (2002). Molecular mechanisms of axon guidance.. Science.

[27] Tessier-Lavigne M, Goodman CS (1996). The molecular biology of axon guidance.. Science.

[28] Hack MA, Saghatelyan A, de Chevigny A, Pfeifer A, Ashery-Padan R (2005). Neuronal fate determinants of adult olfactory bulb neurogenesis.. Nat Neurosci.

[29] Gossen M, Bujard H (1992). Tight Control of Gene-Expression in Mammalian-Cells by Tetracycline-Responsive Promoters.. Proceedings of the National Academy of Sciences of the United States of America.

[30] Schedl A, Ross A, Lee M, Engelkamp D, Rashbass P (1996). Influence of PAX6 gene dosage on development: overexpression causes severe eye abnormalities.. Cell.

[31] Manuel M, Pratt T, Liu M, Jeffery G, Price DJ (2008). Overexpression of Pax6 results in microphthalmia, retinal dysplasia and defective retinal ganglion cell axon guidance.. BMC Dev Biol.

[32] Tyas DA, Simpson TI, Carr CB, Kleinjan DA, van Heyningen V (2006). Functional conservation of Pax6 regulatory elements in humans and mice demonstrated with a novel transgenic reporter mouse.. BMC Dev Biol.

[33] Zhu Y, Guthrie S (2002). Expression of the ETS transcription factor ER81 in the developing chick and mouse hindbrain.. Developmental Dynamics.

[34] Schmidtmer J, Engelkamp D (2004). Isolation and expression pattern of three mouse homologues of chick Rgm.. Gene Expression Patterns.

[35] Haubst N, Berger J, Radjendirane V, Graw J, Favor J (2004). Molecular dissection of Pax6 function: the specific roles of the paired domain and homeodomain in brain development.. Development.

[36] Di Meglio T, Nguyen-Ba-Charvet KT, Tessier-Lavigne M, Sotelo C, Chedotal A (2008). Molecular mechanisms controlling midline crossing by precerebellar neurons.. J Neurosci.

[37] Bloch-Gallego E, Causeret F, Ezan F, Backer S, Hidalgo-Sanchez M (2005). Development of precerebellar nuclei: instructive factors and intracellular mediators in neuronal migration, survival and axon pathfinding.. Brain Res Brain Res Rev.

[38] Mu XQ, Beremand PD, Zhao S, Pershad R, Sun HX (2004). Discrete gene sets depend on POU domain transcription factor Brn3b/Brn-3.2/POU4f2 for their expression in the mouse embryonic retina.. Development.

[39] Erkman L, Yates PA, McLaughlin T, McEvilly RJ, Whisenhunt T (2000). A POU domain transcription factor-dependent program regulates axon pathfinding in the vertebrate visual system.. Neuron.

[40] Pan L, Yang Z, Feng L, Gan L (2005). Functional equivalence of Brn3 POU-domain transcription factors in mouse retinal neurogenesis.. Development.

[41] Liu W, Mo ZQ, Xiang MQ (2001). The Ath5 proneural genes function upstream of Brn3 POU domain transcription factor genes to promote retinal ganglion cell development.. Proceedings of the National Academy of Sciences of the United States of America.

[42] Wang SW, Kim BS, Ding K, Wang H, Sun DT (2001). Requirement for math5 in the development of retinal ganglion cells.. Genes & Development.

[43] Yang Z, Ding K, Pan L, Deng M, Gan L (2003). Math5 determines the competence state of retinal ganglion cell progenitors.. Developmental Biology.

[44] Wagner KD, Wagner N, Vidal VP, Schley G, Wilhelm D (2002). The Wilms' tumor gene Wt1 is required for normal development of the retina.. Embo J.

[45] Krestel HE, Mayford M, Seeburg PH, Sprengel R (2001). A GFP-equipped bidirectional expression module well suited for monitoring tetracycline-regulated gene expression in mouse.. Nucleic Acids Res.

[46] Gotz M, Stoykova A, Gruss P (1998). Pax6 controls radial glia differentiation in the cerebral cortex.. Neuron.

[47] Grindley JC, Davidson DR, Hill RE (1995). The role of Pax-6 in eye and nasal development.. Development.

[48] Marillat V, Sabatier C, Failli V, Matsunaga E, Sotelo C (2004). The slit receptor Rig-1/Robo3 controls midline crossing by hindbrain precerebellar neurons and axons.. Neuron.

[49] Gan L, Xiang M, Zhou L, Wagner DS, Klein WH (1996). POU domain factor Brn-3b is required for the development of a large set of retinal ganglion cells.. Proceedings of the National Academy of Sciences of the United States of America.

[50] Xiang M (1998). Requirement for Brn-3b in early differentiation of postmitotic retinal ganglion cell precursors.. Developmental Biology.

[51] Eng SR, Gratwick K, Rhee JM, Fedtsova N, Gan L (2001). Defects in sensory axon growth precede neuronal death in Brn3a-deficient mice.. Journal of Neuroscience.

[52] Huang EJ, Liu W, Fritzsch B, Bianchi LM, Reichardt LF (2001). Brn3a is a transcriptional regulator of soma size, target field innervation and axon pathfinding of inner ear sensory neurons.. Development.

[53] de Diego I, Kyriakopoulou K, Karagogeos D, Wassef M (2002). Multiple influences on the migration of precerebellar neurons in the caudal medulla.. Development.

[54] Bloch-Gallego E, Ezan F, Tessier-Lavigne M, Sotelo C (1999). Floor plate and netrin-1 are involved in the migration and survival of inferior olivary neurons.. Journal of Neuroscience.

[55] Causeret F, Danne F, Ezan F, Sotelo C, Bloch-Gallego E (2002). Slit antagonizes netrin-1 attractive effects during the migration of inferior olivary neurons.. Developmental Biology.

[56] Causeret F, Hidalgo-Sanchez M, Fort P, Backer S, Popoff MR (2004). Distinct roles of Rac1/Cdc42 and Rho/Rock for axon outgrowth and nucleokinesis of precerebellar neurons toward netrin 1.. Development.

[57] Alcántara S, Ruiz M, De Castro F, Soriano E, Sotelo C (2000). Netrin 1 acts as an attractive or as a repulsive cue for distinct migrating neurons during the development of the cerebellar system.. Development.

[58] Gilthorpe JD, Papantoniou EK, Chedotal A, Lumsden A, Wingate RJT (2002). The migration of cerebellar rhombic lip derivatives.. Development.

[59] Fairhead C, Llorente B, Denis F, Soler M, Dujon B (1996). New vectors for combinatorial deletions in yeast chromosomes and for gap-repair cloning using ‘split-marker’ recombination.. Yeast.

[60] Ben-Arie N, McCall AE, Berkman S, Eichele G, Bellen HJ (1996). Evolutionary conservation of sequence and expression of the bHLH protein Atonal suggests a conserved role in neurogenesis.. Human Molecular Genetics.

[61] Schwab MH, Druffel-Augustin S, Gass P, Jung M, Klugmann M (1998). Neuronal basic helix-loop-helix proteins (NEX, neuroD, NDRF): Spatiotemporal expression and targeted disruption of the NEX gene in transgenic mice.. Journal of Neuroscience.

[62] Ackerman SL, Kozak LP, Przyborski SA, Rund LA, Boyer BB (1997). The mouse rostral cerebellar malformation gene encodes an UNC-5-like protein.. Nature.

[63] Holmes GP, Negus K, Burridge L, Raman S, Algar E (1998). Distinct but overlapping expression patterns of two vertebrate slit homologs implies functional roles in CNS development and organogenesis.. Mechanisms of Development.

[64] Engelkamp D (2002). Cloning of three mouse Unc5 genes and their expression patterns at mid-gestation.. Mechanisms of Development.

