# Supplementary figures and images for: A Novel Approach to Selectively Target Neuronal Subpopulations Reveals Genetic Pathways That Regulate Tangential Migration in the Vertebrate Hindbrain
Source: PLoS Genet. 2011 Jun 16;7(6):e1002099. doi: 10.1371/journal.pgen.1002099 (PMC3116914; doi:10.1371/journal.pgen.1002099)

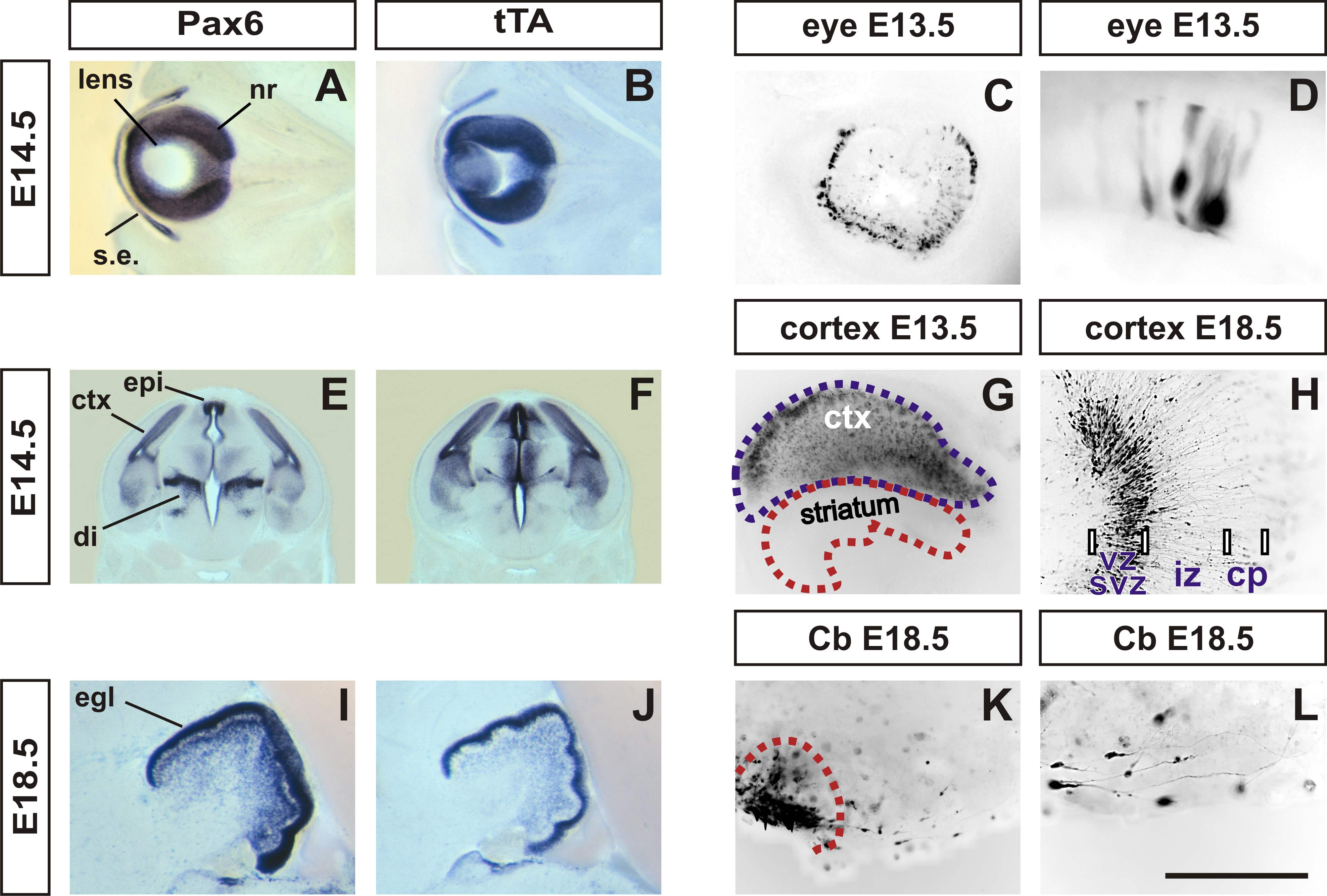

Supplement: Figure S1 — Pax6, tTA and EGFP expression in tg(PAX6-tTA) embryos. (A,B,E,F,I,J) In situ hybridizations of coronal vibratome sections of tg(PAX6-tTA) transgenic embryos. Alternating sections were stained for either Pax6 (A,E,I) or tTA (B,F,J). In the developing eye, Pax6 and tTA are co-expressed in the neural retina (nr), the lens, and the surface ectoderm (s.e.) which generates the future cornea. In the forebrain, both genes are co-expressed in the cortex (ctx), the ventral diencephalon (di), and the epithalamus (epi). In the cerebellum, Pax6 and tTA are co-expressed in granule cells of the external granule cell layer (egl). (C,D,G,H,K,L) Electroporation of EGFP reporter constructs into tg(PAX6-tTA) transgenic embryos. After electroporation tissues were cultured for one or two days on MilliporeCM filters. Reporter gene expression is activated in the developing retina, but not in the surrounding tissue (C); (D) Higher magnification of retinal precursor cells. (G) Electroporation of the telencephalon at E13.5 results in a specific labeling of the Pax6 positive cortex (ctx, blue dotted line), but not of the Pax6 negative striatum (red dots) or other surrounding tissues. (H) Electroporation of the E18.5 anterior telencephalon reveals precursor cells with the typical appearance of radial glial cells; vz, svz, iz and cp denote the ventricular, subventricular, and intermediate zones, and the cortical plate, respectively. (K) In the developing cerebellum EGFP expression is seen in granule cells which leave the transfected region and start to migrate parallel to the cerebellar surface. (L) Higher magnification of (K). Scale bar: 0.6 mm in [A,B]; 1.9 mm in [E,F]; 0.8 mm in [I,J]; 400 µm in [C,H, K]; 1.7 mm in [G]; and 150 µm in [D,L]. (TIF) [file pgen.1002099.s001.tif]

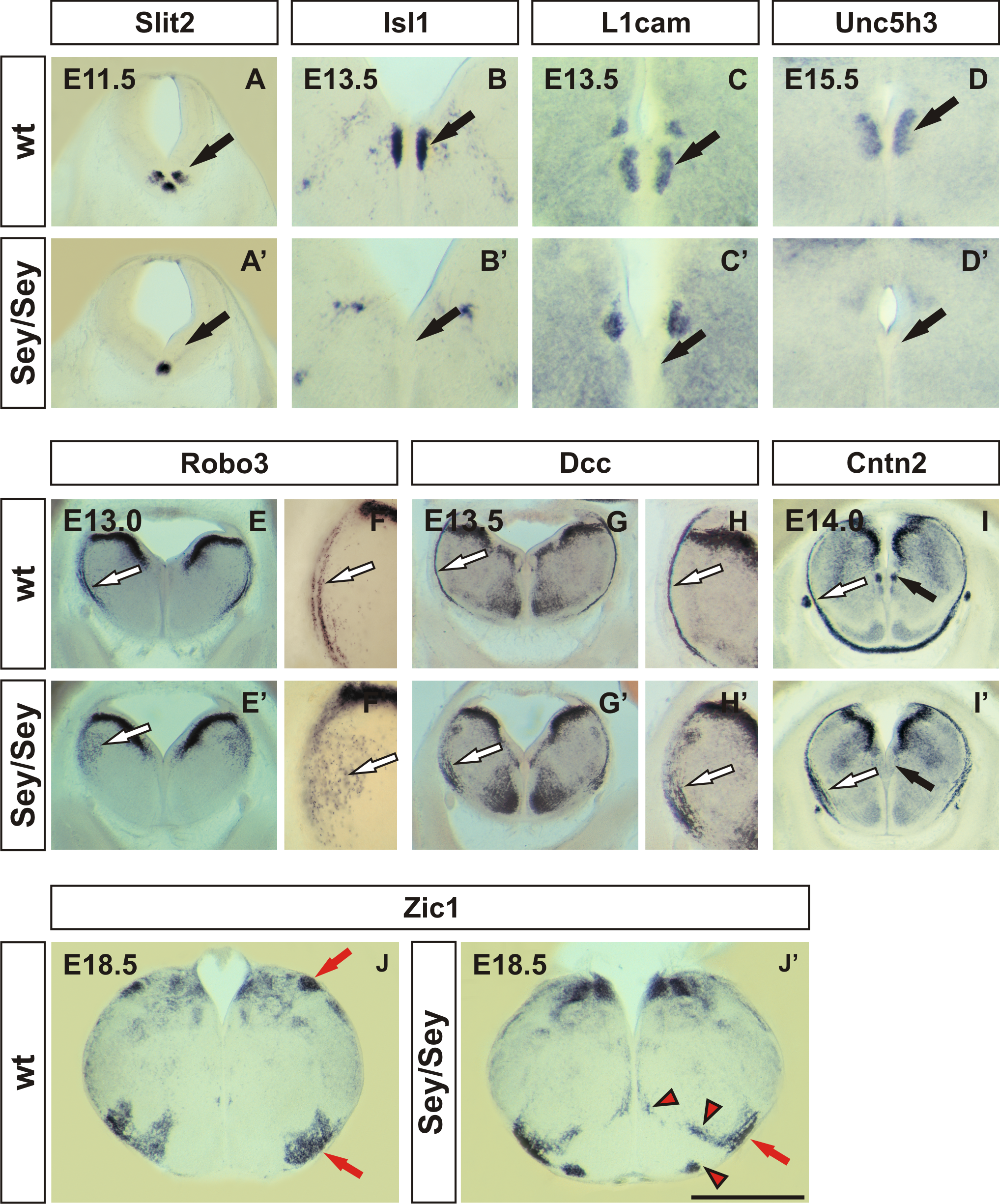

Supplement: Figure S2 — Pax6Sey/Sey Brainstem defects. The hypoglossal nucleus (black arrows) is lost in Pax6Sey/Sey embryos as shown by Slit2, Isl1, L1Cam, and Unc5h3 labelling (A–D). Migrating mms neurons (white arrows) take a submarginal route in Pax6Sey/Sey embryos as shown by Robo3, Dcc, and Cntn2 (also: Tag1) staining (E–I). Zic1 marks mms neurons which ectopically settle in the IO territory of Pax6Sey/Sey embryos (J). Red arrows indicate positions of the ECN and LRN and red arrowheads indicate ectopic settlement of mms neurons in Pax6Sey/Sey embryos. (Scale bar is 0.3mm in [A, A′]; 0.2 mm in [B, B′, C, C′, D, D′, F, F′, H, H′]; 0.9 mm in [E, E′]; 1 mm in [G, G′]; 1.2 mm in [I, I′]; 2 mm in [J, J′].) (TIF) [file pgen.1002099.s002.tif]

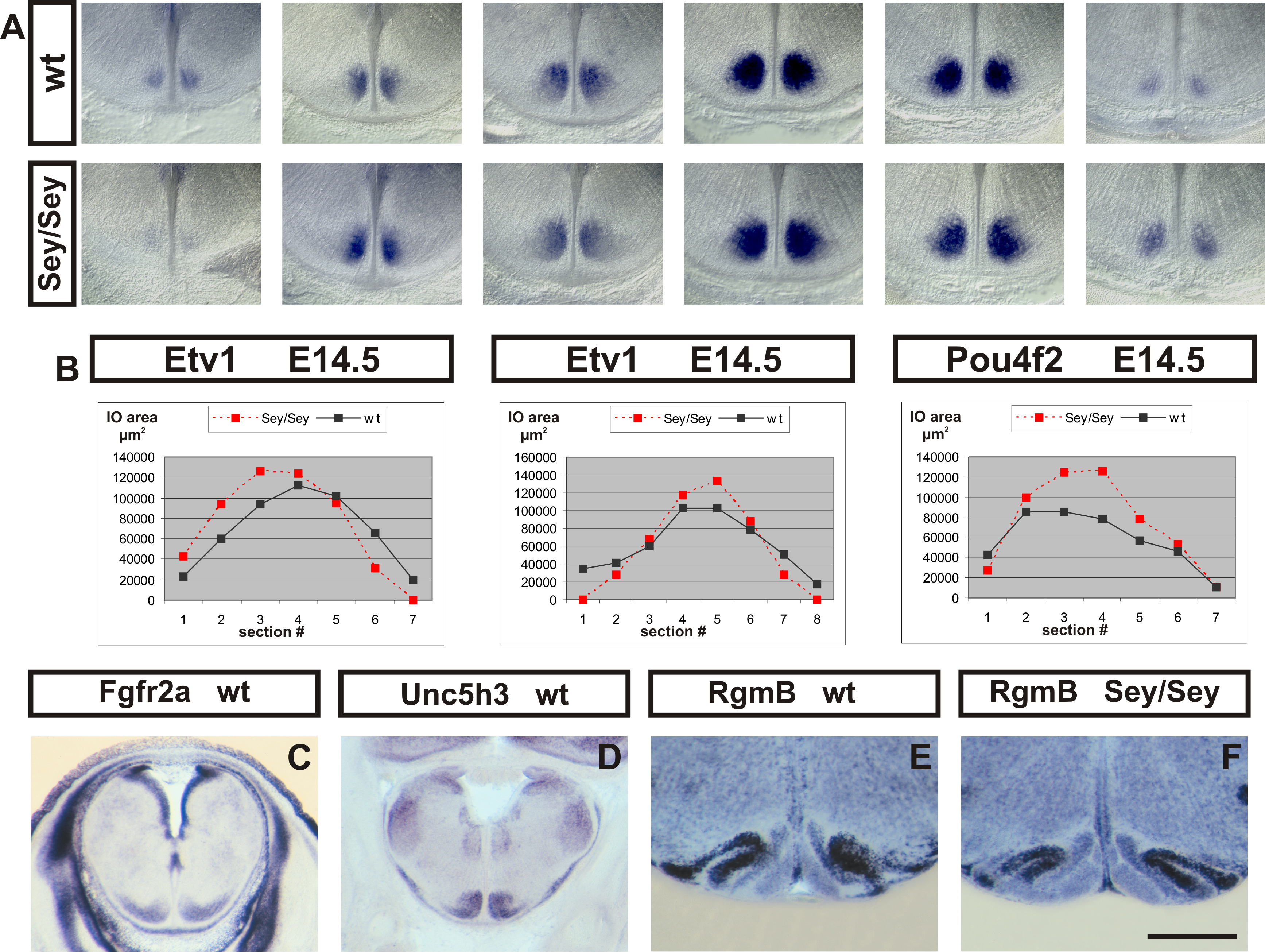

Supplement: Figure S3 — Increased size of the Pax6Sey/Sey inferior olive. (A) Complete series of coronal E14.5 wt and Pax6Sey/Sey brainstem vibratome sections stained with Etv1 by in situ hybridization. (B) Examples of the size determination of the inferior olivary nuclei. Shown are two individual pairs of E14.5 embryos stained with Etv1 and one pair of E14.5 embryos stained with Pou4f2. Note that Etv1 is a specific marker for inferior olivary cells, whereas, Pou4f2 stains mms and inferior olivary neurons and may therefore also include mms neurons that have ectopically migrated into the inferior olivary territory. Values are given in µm2 for the area taken by the left plus right inferior olivary sub-nuclei. (C,D,E,F) In situ hybridization of coronal wt (C,D,E) and Pax6Sey/Sey (F) E14.5 (C,D) and E18.5 (E,F) vibratome sections. Fgfr2a (C) and Unc5h3 (D) label both: migrating mms neurons and IO neurons. RgmB labels equally the dorsal and principal sub-nuclei of the wt and the Pax6Sey/Sey inferior olive suggesting a normal patterning of the mutant inferior olivary nucleus. Scale bar: 0.44 mm in [A]; 0.7 mm in [C,D]; 0.55 mm in [E,F]. (TIF) [file pgen.1002099.s003.tif]

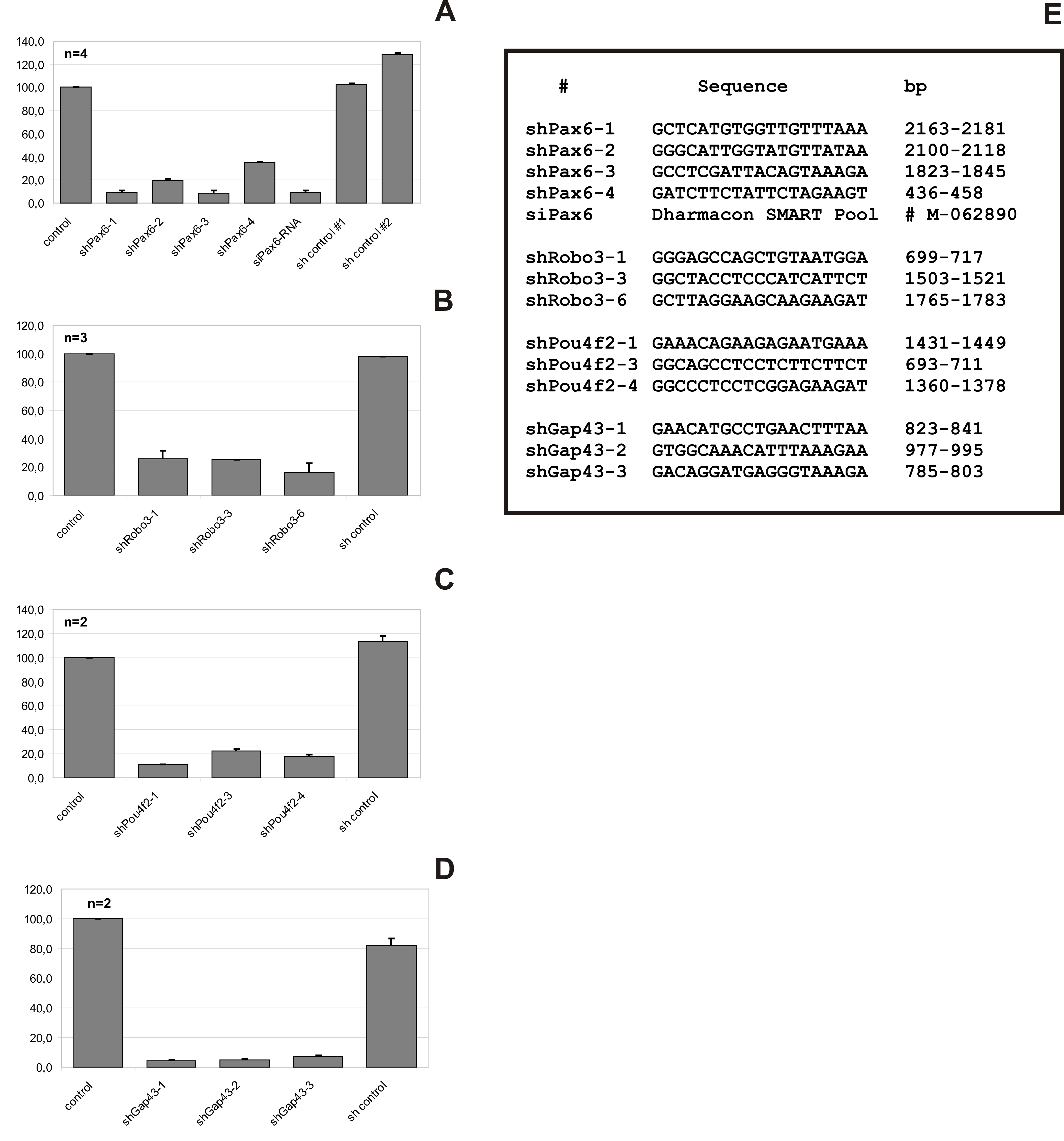

Supplement: Figure S4 — Efficiency of shRNA and siRNA gene knockdown. (A–D) Efficiency of RNA knockdown. The fold repression was determined by the degree of silencing of Renilla luciferase-targeting construct relative to the firefly luciferase control. Averages of two to four individual experiments are shown as indicated. (E) The target sequences of shRNA constructs as indicated by their positions in Pax6 (accession # NM_123627), Robo3 (AF060570), Pou4f2 (S68377), and Gap43 (NM_008083) cDNAs. Dharmacon siRNA SMART pools consist of a pool of 3 individual siRNAs; the sequence is not provided. (TIF) [file pgen.1002099.s004.tif]

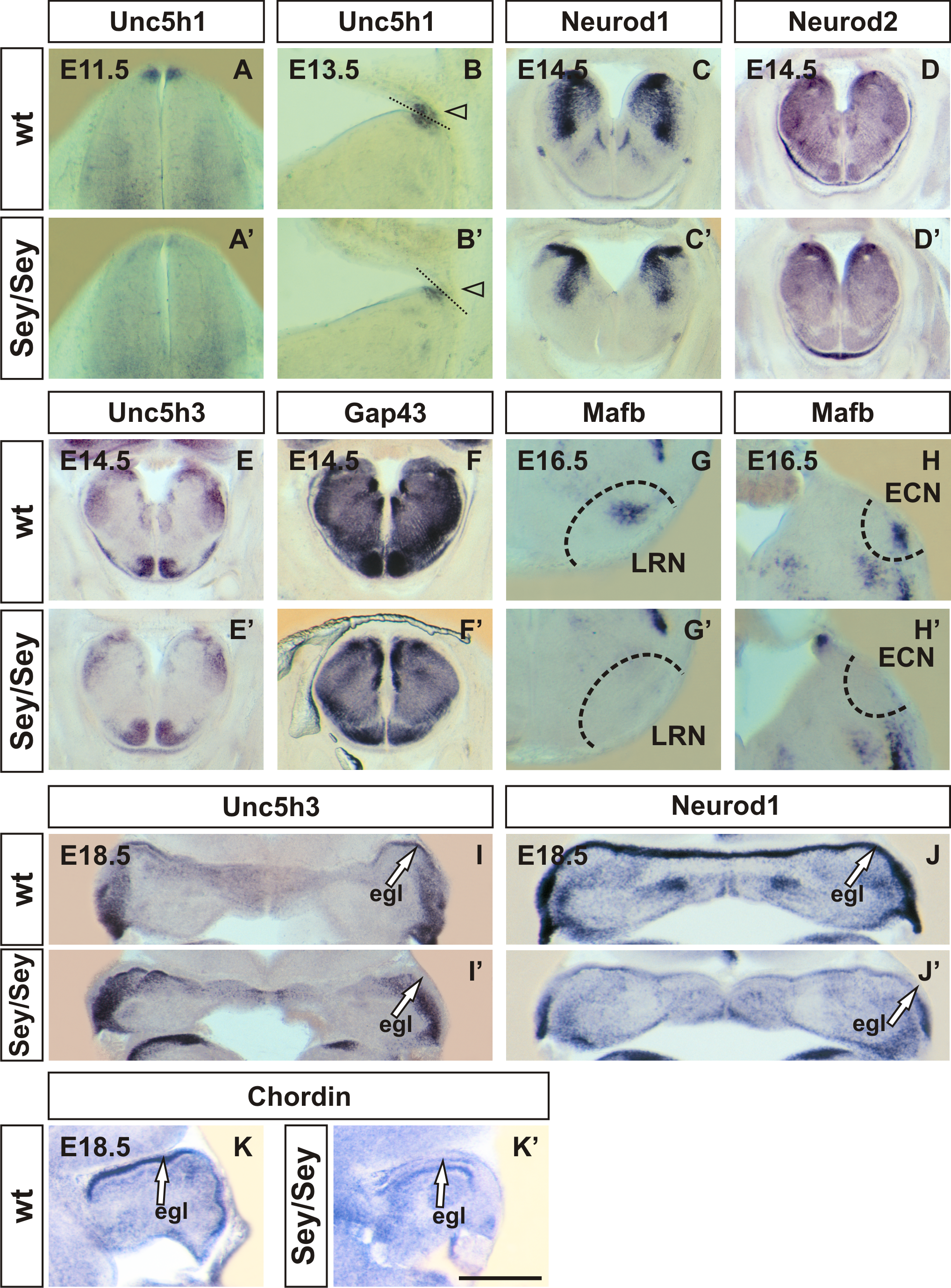

Supplement: Figure S5 — Altered gene expression in the Pax6Sey/Sey hindbrain. Differential expression of putative Pax6 downstream genes in the RL (A,B), the mms (C–H) and the cerebellum (I–K). The dotted line in B, B′ demarcates the boundary between the rhombic lip (open arrowhead) and the remaining alar plate. White arrows in I–K indicate the external granule cell layer (egl). (Scale bar is 0.4 mm in [A, A′]; 0.2 mm in [B, B′]; 0.7 mm in [C, C′, D, D′, E, E′, F, F′, I, I′, J, J′, K, K′]; 0.3 mm in [G, G′, H, H′].) (TIF) [file pgen.1002099.s005.tif]
